# Mannosylated Systems for Targeted Delivery of Antibacterial Drugs to Activated Macrophages

**DOI:** 10.3390/ijms232416144

**Published:** 2022-12-18

**Authors:** Igor D. Zlotnikov, Maksim A. Vigovskiy, Maria P. Davydova, Milan R. Danilov, Uliana D. Dyachkova, Olga A. Grigorieva, Elena V. Kudryashova

**Affiliations:** 1 Faculty of Chemistry, Lomonosov Moscow State University, Leninskie Gory, 1/3, 119991 Moscow, Russia; 2 Medical Research and Education Center, Institute for Regenerative Medicine, Lomonosov Moscow State University, 27/10, Lomonosovsky Ave., 119192 Moscow, Russia; 3 Faculty of Medicine, Lomonosov Moscow State University, 27/1, Lomonosovsky Prosp., 119192 Moscow, Russia

**Keywords:** macrophage uptake, mannose receptor, pharmacokinetics, adjuvant, drug delivery system

## Abstract

Macrophages are a promising target for drug delivery to influence macrophage-associated processes in the body, namely due to the presence of resistant microorganisms in macrophages. In this work, a series of mannosylated carriers based on mannan, polyethylenimine (PEI) and cyclodextrin (CD) was synthesized. The molecular architecture was studied using FTIR and ^1^H NMR spectroscopy. The particle size, from small 10–50 nm to large 500 nm, depending on the type of carrier, is potentially applicable for the creation of various medicinal forms: intravenous, oral and inhalation. Non-specific capture by cells with a simultaneous increase in selectivity to CD206+ macrophages was achieved. ConA was used as a model mannose receptor, binding galactosylated (CD206 non-specific) carriers with constants of the order of 10^4^ M^−1^ and mannosylated conjugates of 10^6^–10^7^ M^−1^. The results of such primary “ConA-screening” of ligands are in a good agreement in terms of the comparative effectiveness of the interaction of ligands with the CD206+ macrophages: non-specific (up to 10%) absorption of highly charged and small particles; weakly specific uptake of galactosylated polymers (up to 50%); and high affine capture (more than 70–80%) of the ligands with grafted trimannoside was demonstrated using the cytometry method. Double and multi-complexes of antibacterials (moxifloxacin with its adjuvants from the class of terpenoids) were proposed as enhanced forms against resistant pathogens. In vivo pharmacokinetic experiments have shown that polymeric carriers significantly improve the efficiency of the antibiotic: the half-life of moxifloxacin is increased by 2–3 times in conjugate-loaded forms, bio-distribution to the lungs in the first hours after administration of the drug is noticeably greater, and, after 4 h of observation, free moxifloxacin was practically removed from the lungs of rats. Although, in polymer systems, its content is significant—1.2 µg/g. Moreover, the importance of the covalent crosslinking carrier with mannose label was demonstrated. Thus, this paper describes experimental, scientifically based methods of targeted drug delivery to macrophages to create enhanced medicinal forms.

## 1. Introduction

Macrophages play an important role in both innate and acquired (humoral and cellular) immune responses. Their specialized derivatives, dendritic cells, have the unique ability to induce T- and B-lymphocytes, while macrophages affect a range of immune responses by recognition, capture, purification and transport. They attract hematopoietic cells to local foci of inflammation and immunity and regulate their activity [1]. Macrophages phagocytize foreign material and cellular-tissue detritus, stimulate and regulate the immune response, induce an inflammatory reaction, participate in reparative processes and exchange of extracellular matrix components [2,3,4]. The effect on macrophages can be classified into three groups according to the mechanism: activation of macrophages from an inactive state in M1 (pro-inflammatory) or M2 (pro-tumor or anti-inflammatory) [5]; repolarization of M2 in M1 to reduce the rapid response of the immune system [6,7]; accumulation of antibacterial substances in macrophages against microorganisms “hidden in the reservoir” [8]. Due to the possibility of the multipoint control of macrophages, the targeted delivery of drugs to immune cells opens up numerous prospects in the treatment of a wide range of diseases, including respiratory tract diseases [9,10], neurodegenerative [11], autoimmune [3,12,13,14] and cancer diseases [15,16]. The variety of functions determines the presence of a large number of receptors in macrophages, with the help of which the drug delivery system can perform a given function.

In this paper, the aspect of the targeted delivery of antibacterial drugs to macrophages by targeting mannose receptors is considered [9,10,14,17,18,19,20]. The CD206 mannose receptor is of greatest interest, which is involved in the recognition of pathogens due to the interaction of protein-binding domains with oligosaccharide patterns of microorganisms (*Candida albicans*, *Pneumocystis carinii*, *Leishmania donovani*, *Mycobacterium tuberculosis*, *Klebsiella pneumoniae*, etc.) [2,21]. The CD206 receptor mainly allows for targeting activated macrophages, in which resistant and dormant infections can accumulate. Selectivity toward micro-organisms is achieved due to the specificity of CD206 to mannose, fucose and N-acetylglucosamine residues, which often cover the surface of pathogen cells, unlike mammals [17,18,22,23,24]. For example, lipoarabinomannan is a major lipoglycan on the surface of Mycobacterium tuberculosis; it is recognized by CD206, which participates in the phagocytosis of the bacterium while activating the complement system and signaling pathways [25]. However, the phagocytic bacterium is able to lower the pH inside the macrophage, thereby preserving itself intact and in a protective shell [8]. Earlier, we outlined the problem of the emergence of resistant and intractable strains of pathogens [25]. The two main vectors of the fight against this kind of infection are as follows: (i) the use of adjuvants (eugenol, apiol, other terpenoids), antibiotic enhancers that inhibit efflux and increase the permeability of the bacterial membrane [26,27,28,29,30,31,32,33,34,35]; (ii) the use of targeted drug delivery systems to macrophages in order to increase their local content and circulation time [9,10,14,16,17,18,22,36,37,38,39,40,41,42,43,44,45,46,47,48,49,50,51,52,53].

The aim of this work is to develop a combined formulation based on 4th-generation fluoroquinolone moxifloxacin (MF) loaded into mannosylated containers as a target delivery system and enhanced with adjuvants (EG [29,54,55,56,57,58,59,60,61,62,63,64,65,66], menthol, and safrole [33,67,68]) with prerequisites for overcoming multidrug resistance. The adjuvant effect was recently studied in detail: it was shown that EG can significantly increase the effect of the antibiotic (levofloxacin and moxifloxacin—MF) and reduce MIC by three times [25]. In this work, we continued to study enhanced formulations based on fluoroquinolones, while the main attention was focused on the effect of drug delivery systems on MF uptake by macrophages and the pharmacokinetics of complex formulations. Mannosylated particles are much more efficiently absorbed by macrophages than unmodified polymers [37,51,53,69]. Moreover, it has been shown that the effectiveness of drug delivery systems is higher if the following requirements are met [53]: the carrier has neither a large nor too small molecular weight (5–15 kDa), an average degree of mannosylation (a larger number of Man residues can sterically interfere with binding to CD206) and the distance between mannose clusters is 5–6 nm. Previously, we synthesized a wide range of mannosylated delivery systems: chitosans with mannose clusters on spermine [45], mannosylated polyethylenimines, crosslinked cyclodextrin meshes [70] and multicomponent conjugates—which allowed optimizing the molecular container in terms of drug loading efficiency and high affinity to the model mannose receptor ConA. The use of this primary screening model simplified and significantly accelerated the creation of optimal carriers [17,22].

In this paper, mannan grafted with cyclodextrin and modified polyethylenimines (PEIs) are considered as promising carriers of drugs to macrophages. Oligomannoside fragments used in the work form strong complexes with mannose receptors. Linear mannose was chosen as the label of average affinity and a non-specific carbohydrate modification—linear galactose. Therefore, here we present correlations of data on the affinity of the model receptor to the «target» label and molecular architecture of the conjugate with the absorption of particles by CD206+ macrophages in comparison with CD206- cells and pharmacokinetic parameters of the loaded drug and the accumulation in the lungs of rats. Thus, this paper discusses the relevance of using the ConA model in the initial stages of the development of mannosylated systems and pilot trials of complex formulations of fluoroquinolones with its adjuvants (terpenoids) as promising drugs for combating drug resistance of pathogens localized in macrophages.

## 2. Results and Discussion

### 2.1. Synthesis and Characterization of Ligands

The investigation of ligands of different molecular architecture, molecular weight and carbohydrate conjugate labels with different affinity to mannose receptors is due to the need to create optimal delivery systems and to clarify the trends and influence of each of the factors on the CD206+ macrophage uptake. Three series of ligands were studied: mannans grafted with cyclodextrins; polyethylenimines with grafted CDs and target labels; star-shaped CDs with spermines.

**Figure 1:**
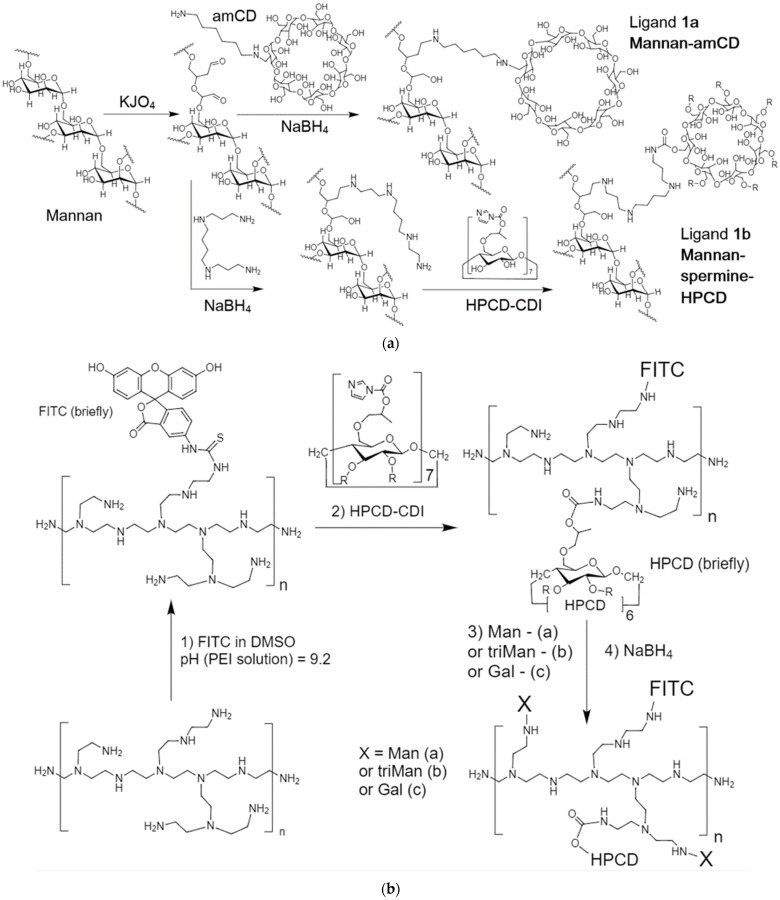

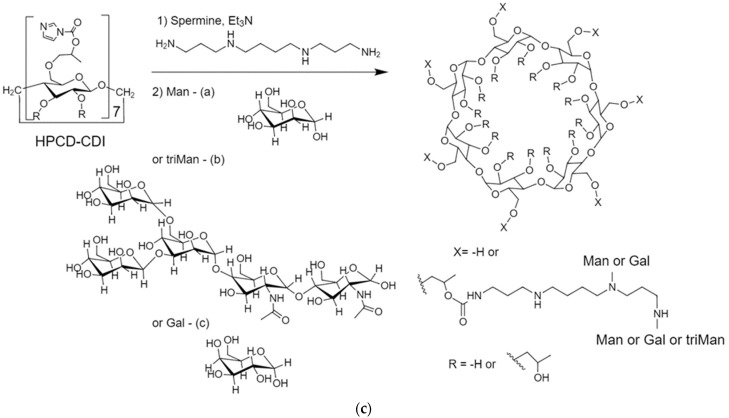
Scheme of synthesis of (**a**) mannan-amCD and mannan-spermine-HPCD, (**b**) FITC-labeled HPCD-PEI1.8 with grafted Man or Gal or triMan, (**c**) FITC-labeled star-shaped HPCD-spermine with grafted Man or Gal or triMan.

**Figure 2:**
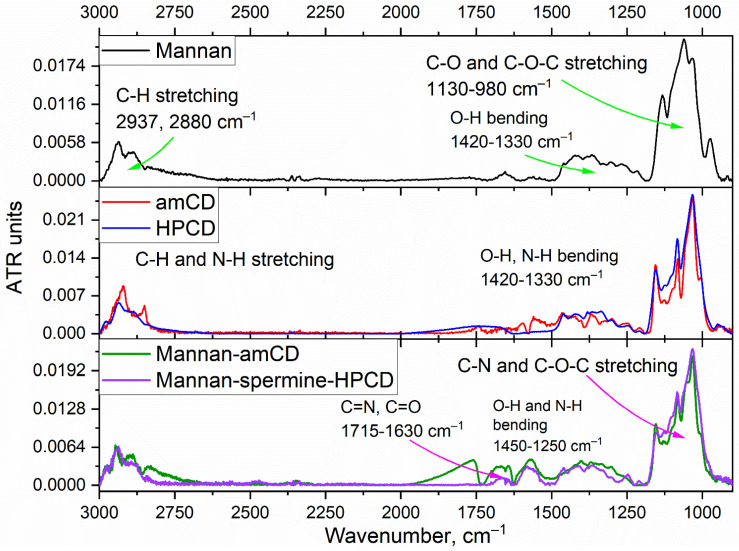
Fourier transform infrared spectra of mannan, amCD, HPCD, mannan-amCD and mannan-spermine-HPCD. T = 22 °C.

**Figure 3:**
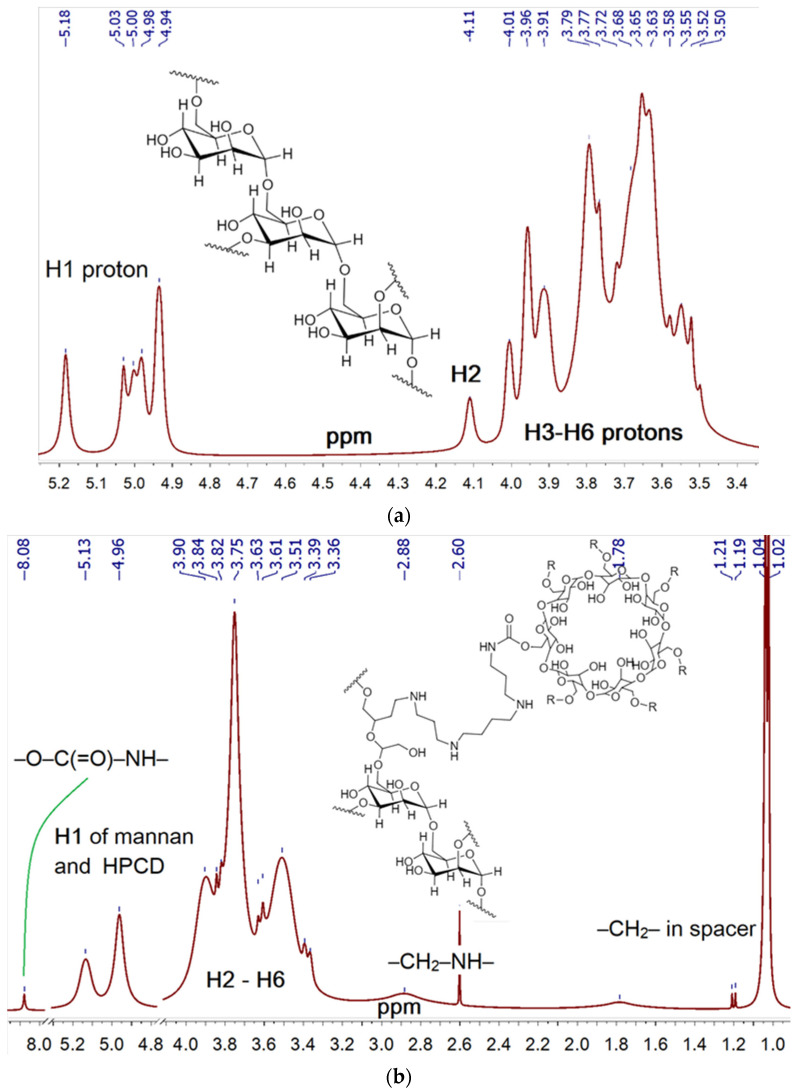
^1^H NMR spectra of (**a**) mannan, (**b**) mannan-spermine-HPCD. D_2_O, 400 MHz.

The reactions underlying the synthesis of conjugates 1–3 are as follows (Figure 1): reductive amination of aldehyde groups [52,71,72,73], oxidation of pyranose cycles by periodate [74,75,76,77,78], activation of hydroxyl groups of cyclodextrin CDI followed by crosslinking with NH_2_– and OH– groups [70,79,80,81] and nucleophilic addition by thiocarbonyl group (FITC labelling). Ligands 1a, 1b were synthesized on the basis of mannan into two–three steps (Figure 1a). The periodate-oxidation of mannan (i.e., conversion of secondary hydroxyl groups into aldehydic functionality) is confirmed by the appearance of the characteristic -C = O band at 1640–1780 cm^−1^ in FTIR spectra (Figure S1); by shifting the absorption bands of the valence oscillations of the CH_2_ bonds from 2937 and 2880–2907 to 2982 and 2938 cm^−1^, respectively; and by a significant narrowing and shifting of the peaks of the valence oscillations of the C-O-C and C-O bonds in carbohydrate—1131, 1062 and 1035 cm^−1^ are transformed into 1086 and 1043 cm^−1^ due to the disclosure of part of the mannopyranose cycles and changes in the microenvironment of these groups. The subsequent crosslinking of -C = O groups of oxi-mannan with amino groups of spermine (Figure 1a) with the formation of a Schiff base leads to an increase in the intensity of the peak of 1630–1720 cm^−1^ corresponding to the oscillations of C = N and C = O bonds (Figure 2), the integral area of which decreases with borohydride reduction. Additionally, HPCD is attached to mannan–spermine to obtain ligand 1b due to the reaction of activated CDI hydroxyl groups of cyclodextrin with amino groups of spermine; in the FTIR spectrum, the characteristic peak 1630 cm^−1^ corresponding to –O–C(=O)–N is amplified. The structure of conjugate 1 was confirmed with NMR spectroscopy (Figure 3a). Characteristic chemical shifts of protons H1 (5.0–5.2 ppm), H2 (4.0–4.2 ppm) and H3–H6 (3.5–4.0 ppm) are observed in the mannan ^1^H NMR spectrum. The formation of mannan conjugate with cyclodextrin through the spacer spermine is confirmed by the presence of characteristic shifts (Figure 3b): –CH_2_– (1.0, 1.2 and 1.8 ppm), –NH– (2.6–2.8 ppm) and –O–C(=O)–NH– (8.1 ppm).

The synthesis of conjugates **2** and **3** is performed similarly to the previously described protocol (Figure 1b,c) [70]. Additionally, a fluorescent label and three types of carbohydrate labels of different affinity, quantitatively normalized for the saccharide link, were introduced for a relevant macrophage uptake comparison. FITC labelling on PEI is accompanied by the formation of –NH–C(=S)–NH–bonds. The characteristic peak of the thiocarbonyl group oscillations in the FTIR spectra (Figure S2) is 1215 cm^−1^. The synthesis of the molecular container is carried out by activating the hydroxyl groups of HPCD with carbonyldiimidazole, followed by nucleophilic substitution with amino groups of spermine or PEI. Spermine reacts with HPCD in a ratio of 1:1, and about 2–10 HPCDs are grafted on PEI, depending on the initial ratio of HPCD:PEI. The formation of the –O–C(=O)–NH– crosslinking is confirmed by the absorption fields in the FTIR spectra: 1536–1515 and 1700–1800 cm^−1^ (Figure S2). The introduction of carbohydrate labels based on monomeric mannose, galactose and trimannoside (Figure 1) is based on the formation of a Schiff base between the amino group of the conjugate and the aldehyde group of the reducing saccharide: C–O–C, O–H and C–N–C bonds in the FTIR spectra in the region of 950–1100 cm^−1^. Based on FTIR and NMR spectroscopy data (integral peak intensities) and initial component ratios, the average component ratios in conjugates were determined (Table 1). Ligands of the 1st series are mannan (46 kDa) grafted with 10–15 CD tori, which is a compromise between the parameters of drug capacity and affinity for mannose receptors. Ligands of the **2**nd series are light branched polyethylenimines with grafted 2–5 CD tori, and carbohydrate labels. Ligands of the **3**rd series are cyclodextrin in the form of a star with four grafted spermines on which FITC and saccharide residues are attached. Physico-chemical parameters of synthesized platforms are presented in Table 1.

The hydrodynamic size (Figure S3, Table 1) of the particles was characterized by nanoparticle tracking analysis (NTA). Polymeric conjugates of the first and second series based on PEI or mannan have an average size of about 120–180 nm, which gives two advantages: the possibility of creating an inhaled form of the drug to reduce the stress on the body and effective capture of large particles by macrophages. Star-shaped cyclodextrins are half the size of the order of 70–80 nm, which may exceed the previous particles in a low immune response.

ζ-potential (Figure S4, Table 1) of conjugates was measured using DLS. It is believed that positively or negatively charged (>10 mV) particles interact better with surface proteins or polysaccharides of cell membranes [82,83], which determines effective penetration—what is typical for the star-shaped CD conjugates of the 3rd series. The effect of surface charge on the specificity of cell capture is studied in this paper. Indeed, macrophages non-specifically capture all anionic conjugates, while specificity is achieved for particles whose charge is low (for more information about macrophages, see the corresponding section).

In addition to the size and charges for pharmaceutical preparations, the homogeneity of the obtained ligands is also important. The polydispersity indexes (PDI) determined by the DLS method (Table 1) of synthesized conjugates show quite acceptable characteristics in terms of homogeneity. The relative homogeneity of the samples can also be judged from the zeta-potential distributions of the particles (Figure S4), which are represented by a single peak.

The purification and homogeneity of samples were analyzed by HPLC. The chromatograms are shown in Figure S5. For ligands **1b**, **2b** and **3b**, retention times are 3.1, 2.3 and 3.0 min (dead time is 1.7 min). Polymers are eluted from the column by one main peak; however, the peaks are rather blurred to the right due to some polydispersity of the samples. Therefore, for further experiments, fractions were collected that correspond to the maximum peaks on the chromatograms ±0.5 min by retention time.

### 2.2. Ligand Affinity to the Mannose Receptor on the ConA Model

**Figure 4:**
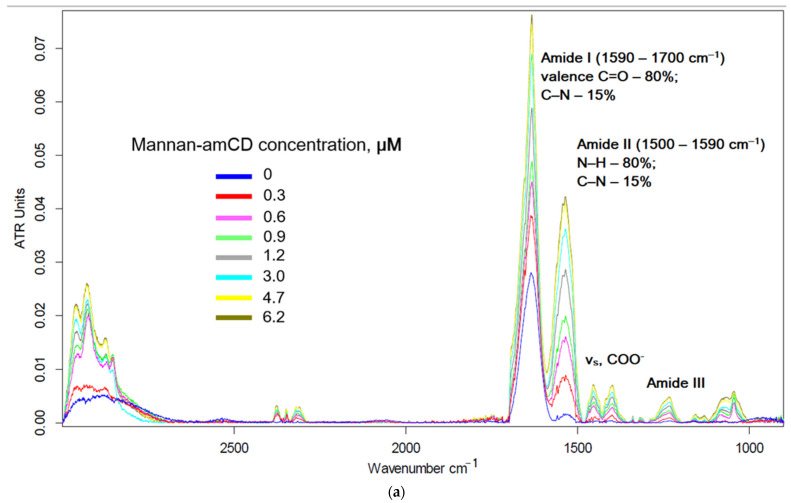

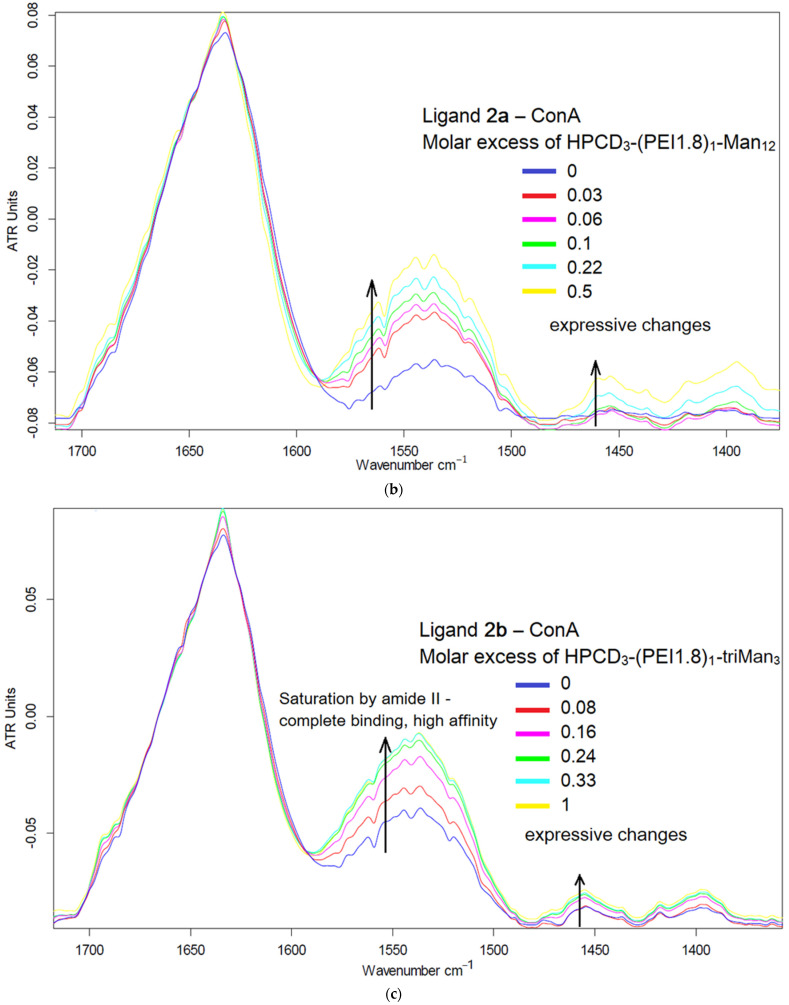

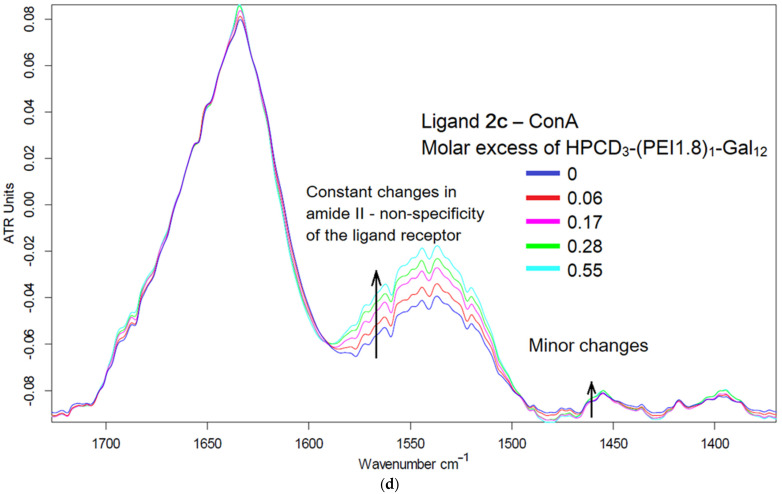
Fourier transform infrared spectra of ConA and ConA complexes with (**a**) mannan-amCD, (**b**) ligand 2a, (**c**) ligand 2b and (**d**) ligand 2c. C_0_(ConA subunit) = 0.1–0.15 mM. Natrium–phosphate buffer solution (0.02 M, pH 6.4). C(Ca^2+^) = C(Mn^2+^) = 1 mM. T = 22 °C. Ligand spectra were subtracted as background.

Model proteins are often used to screen affinities of receptor–ligand interactions. In this paper, ConA was selected as a model of mannose receptors of CD206+ macrophages, for which high similarity with CD206 was previously shown [17,22,45]. It is convenient to study protein–ligand affinity by FTIR spectra that are sensitive to the fine structure of the ligand-binding site [70]. Three main absorption bands are observed in the FTIR spectra of ConA (Figure 4): amide A (2800–3000 cm^−1^), amide I (1600–1700 cm^−1^) and amide II (1490–1600 cm^−1^). It is known that Asn14, Asp16, Arg222 and several glycine and tyrosine residues are involved in the process of complexation of ConA with ligands [22,84,85]. Therefore, when the ligand is bound by the active site of the protein, changes occur in the microenvironment of peptide bonds, side radicals and non-covalent interactions (hydrogen bonds, van der Waals and electrostatic interactions) are formed, which is reflected in the FTIR spectra (Figure 4) [70]. The intensity of the above-mentioned peaks increases. Amide III (~1250 cm^−1^—ν_s_(C-N), δ(N-H)) and band at 1400 cm^−1^ (ν_s_(COO^−^)) also become more expressive.

It is believed that the intensity of the amide I band correlates with the protein concentration [86]; therefore, normalized spectra allow for comparing changes in the amide II-III regions with each other when binding non-specific, specific and high affinity ligands to ConA (Figure 4b–d). The intensity of amide II in the case of galactose-labeled ligand **2** (Figure 4d) varies linearly (not the Hill model) and weakly depends on the concentration of galactosylated molecules, since the interaction of ConA with galactose is non-specific (K_d_ > 10^−2^M); therefore the ligand itself, even due to many point interactions, cannot bind firmly to ConA: −lg K_d_ 3–3.3 (Table 1). At the same time, the interaction of the mannose-labeled ligand with ConA leads to a hyperbolic dependence of the degree of formation on the concentration of the ligand (Figure S6) due to the rather strong binding of the protein to the ligand (−lg K_d_ 5.3–5.4). The conjugate with the trimannose label (**2b** and **3b**) interacts in ConA in a highly affine manner (−lg K_d_ 6–6.6), which leads to a rapid saturation of changes in the amide II region (Figure 4c). An additional visual criterion of ligand–receptor affinity is the 1460 and 1400 cm^−1^ bands, changes in which are observed only for mannosylated (highly specific) ligands (Figure 4b–d). Thus, we have discovered a criterion for the specificity of receptor–ligand binding in IR spectroscopy—almost as a “neural network” screening.

Complexation with ligands leads to changes in the secondary structure of ConA (Figure S7). Based on the spectral deconvolution data (curve-fit; according to the literature data on the correlation of peaks and the molar absorption coefficient [87,88,89,90,91]) of amide I peak in the ConA FTIR spectrum when binding to the ligand, it was shown that the content of β-sheet increases from 46% to 70–80% (excluding intermolecular aggregation) with a simultaneous significant decrease in the content of disordered structures from 23% to 4–7%, and a slight decrease in the content of beta-turns. In addition, when the ligand binds, the protein is aggregated relative to the native form by 30–40% according to the intensity of peaks corresponding to interglobular interactions (1624, 1691, 1696 cm^−1^) [92,93].

Table 1 shows the values of the dissociation constants of ConA complexes with ligands. Mannan-based conjugates form one of the strongest complexes with protein. The ligands of the **2**nd group are generally comparable with the **3**rd in affinity; however, the location of the triMan volume label is more optimal on the HPCD-PEI1.8 polymer chain than on the star-shaped CD. However, the delivery system must meet a number of requirements in addition to affinity, which is described below.

### 2.3. Loading of Moxifloxacin and Adjuvants into Mannosylated Particles

Fluoroquinolone moxifloxacin was chosen as the main active component due to its wide spectrum of action and applicability for the treatment of respiratory tract diseases. Targeted drug delivery and combination with adjuvants will increase the pharmacological properties of MF. Adjuvants were studied: EG, menthol, safrole and their mixture—we showed synergistic effect with fluoroquinolones against *E. coli* and *B. subtilis* [33,70]. In this section, the physicochemical parameters of MF and its adjuvants loading into molecular containers are considered.

**Figure 5:**
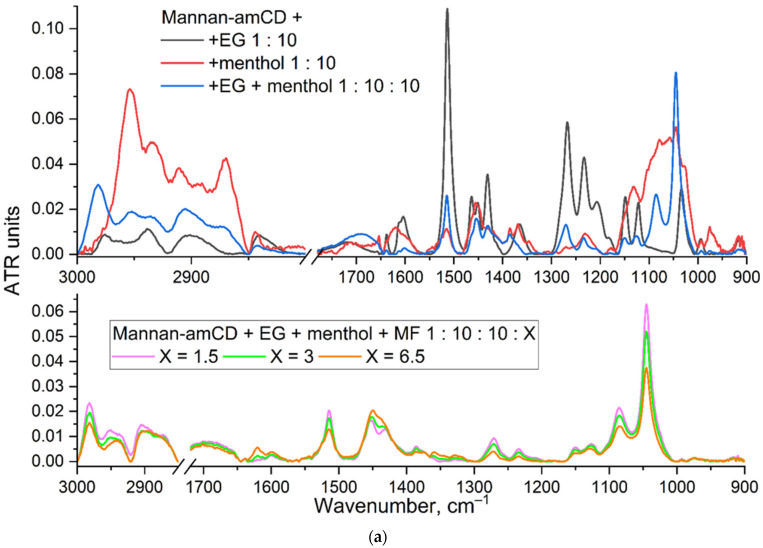

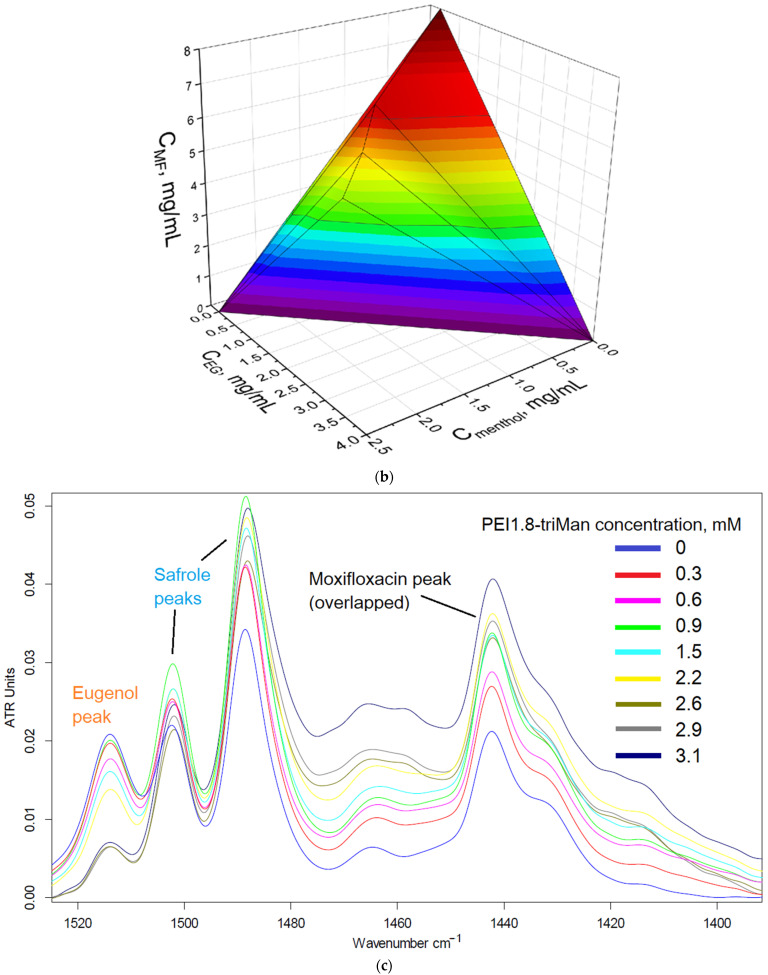
(**a**) Fourier transform infrared spectra of Mannan-amCD complexes with EG, menthol, their mixture and triple formulations MF + EG + menthol with mannan-amCD. The mass ratios are indicated (*w*/*w*). (**b**) Diagram of the distribution of components in the complex formulation mannan-amCD with MF + EG + menthol. (**c**) Aromatic C–C region in FTIR spectra of four components loaded into a molecular container at once: moxifloxacin 0.9 mM, eugenol 17 mM, safrole 12 mM and menthol 12 mM. T = 22 °C. Phosphate buffer solution (0.02 M, pH 6.4). T = 22 °C. Ligand spectra were subtracted as background.

Figure 5a shows the FTIR spectra of mannan-amCD particles (mannan grafted with CD) loaded with EG, menthol and their mixture, as well as the complex triple formulation MF + EG + menthol. EG is characterized by the following FTIR peaks (Figure S8a): the main band is 1513–1518 cm^−1^, corresponding to vibrations of C–C bonds of the aromatic system, 1600–1620 cm^−1^—vibrations of C = C of the allyl group, 1430–1360 cm^−1^ O-H bending and 1300–1100 cm^−1^ C-O stretching. Safrole is characterized by the following peaks in the FTIR spectrum, similar to the EG spectrum (Figure S8b): the main bands are 1502, 1488 and 1443 cm^−1^, corresponding to vibrations of C–C bonds of the aromatic system; 1607 and 1640 cm^−1^—vibrations of C = C of the allyl group; 1300–1100 cm^−1^ C-O stretching.

The intensity of the adjuvant (EG, safrole and menthol) peaks is sensitive to the interaction with the molecular container. There are two parallel processes going on (Figure S8): (1) the transition of adjuvants from the oil fraction to the water-soluble phase in the form of a complex with a molecular container and (2) the inclusion of aqua-phase adjuvant molecules into the CD cavity or interaction with the polymer grid. The first process of dissolution leads to an increase in the intensity of peaks in the FTIR spectrum, and the second process of complexation leads to the quenching of peaks due to the shielding of functional groups and their involvement in noncovalent interactions with the ligand [33].

The FTIR spectrum of menthol (Figure 5a) differs from the EG and safrole spectra and is characterized by peaks: the band at 2953 cm^−1^ corresponds to the asymmetric stretching vibrations of –C–H, –CH_3_, and –CH_2_ groups [94,95]; 2921 cm^−1^ and 2870 cm^−1^—asymmetric and symmetric stretching oscillations –C–H, –CH_2_; 1455 cm^−1^—deformation vibrations of CH_2_ and CH_3_ groups; the spectral band at 1246 cm^−1^ and 1045 cm^−1^ may be assigned to the –C–O stretching vibrations or –CH_2_– deformation vibrations and –C–O stretching vibrations, respectively [94,95,96].

FTIR spectra of MF alone and MF–ligand complexes show C = O stretching at 1621 cm^−1^, aromatic C = C stretching at 1515 and 1448 cm^−1^, carboxylic acid C = O stretching at 1715 cm^−1^, C–N stretching at 1320–1380 cm^−1^ and stretching C–F at 1188 cm^−1^. The formation of non-covalent MF complexes with ligands is accompanied by a decrease in the intensity and a change in the shape of these peaks (Figure 5a). Confirmation of the change in the microenvironment of the quinolone structure of MF is the shift of the peak from 1448 to 1454 cm^−1^.

The characteristic peaks of all synergistic components EG + menthol + MF (Figure 5b) are presented in the FTIR spectra of the complex triple formulation (Figure 5c). The content of loaded MF and adjuvants in a molecular container was calculated by sequentially subtracting the spectra of individual components. The maximum capacity for booster substances is about 4 mg/mL; for MF, it is 8 mg/mL due to stronger interactions of fluoroquinolones with CD [33]. However, the most optimal formulation contains 1 mg/mL of adjuvants and 5–6 mg/mL of MF, respectively, since, in this case, the optimum loading and antibacterial action will be achieved [33].

The inclusion of MF in conjugates is confirmed with NMR spectroscopy. Figure S9 shows the ^1^H NMR spectra of MF alone and MF loaded into mannan-spermine-HPCD. As earlier reported [25], the interaction of MF with a polymer chain leads to induced shifts toward a strong field, which corresponds to an increase in the hydrophobicity of the microenvironment of drug molecules, which indicates the successful encapsulation of MF into a molecular container. The inclusion of an apolar fragment of the MF molecule into the host hydrophobic cavity of HPCD induced a shielding of the inner protons of the glucose units of HPCD, namely, H3 and H5, whereas the protons on the exterior of the torus (H1, H2 and H4) were relatively unaffected; this was previously shown in systems where CD formed inclusion complexes with Lev, EG [33], atropine, nitrobenzene and nicardipine [97,98,99,100,101].

The protons MF 30, 31 and 38 of the piperidine and cyclopropane rings are characterized by strong shifts toward an upfield (Figure S9). Aromatic protons, on the contrary, are characterized by a shift toward a weak field, which is probably explained by the formation of stacking interactions between quinolone fragments of MF in the case of simple MF (Figure S9). However, at the same time, relative to the literature data [102,103], MF is characterized by shifts toward a strong field, which confirms the inclusion of MF in HPCD cavities. The disparity in the chemical shifts of the protons of simple MF is probably related to the used form of MF (lyophilizate/micronized particles).

### 2.4. Capture of Particles by Target CD206+ Macrophages

The purpose of the experiment is studying the influence of molecular architecture and ligand parameters on macrophage absorption. So, we compared molecular containers (proposed drug delivery systems) by their ability to be phagocytized by macrophages, depending on the molecular architecture (linear, star-shaped), the type of base (PEI, CD or mannan) and the “address label” (galactose—control, linear mannose and trimannoside). For the relevance of the comparison, polymers with the same number of FITC labels and the same number of carbohydrate residues were synthesized (Man and Gal—for 1, triMan—for 3–4). The second important aspect is whether the ConA model is relevant and if the data on the affinity of ligands correlate with macrophage uptake and its selectivity.

**Figure 6:**
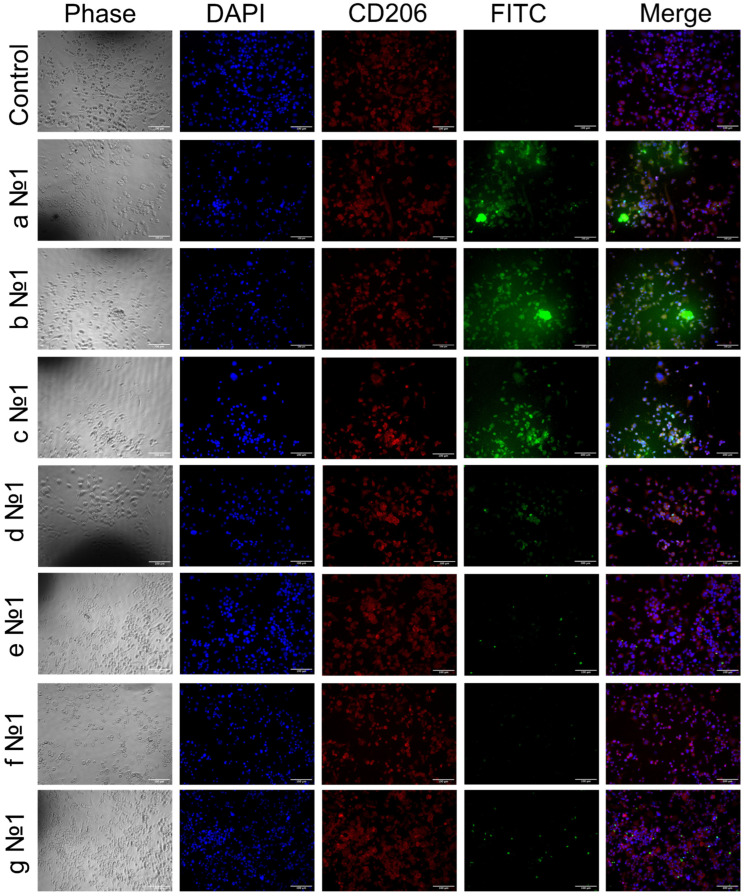
Immunocytochemical evaluation of CD206 in THP-1 derived macrophage-like cells (red channel). Phagocytosis assay with polymers (according to Table S1) at concentration N^o^1 conjugated with a FITC fluorescent label after incubation for 40 min (green channel). Two types of polymers: HPCD-PEI1.8 and HPCD-spermine, modified with mannose (**a**,**e**), trimannose (**b**,**f**) or galactose (**c**,**g**), respectively, and one sample HPCD-spermine modified with mannan (**d**). Phase contrast microscopy, fluorescent microscopy, blue channel—nuclei stained with DAPI. Scale bar, 100 μm.

**Figure 7:**
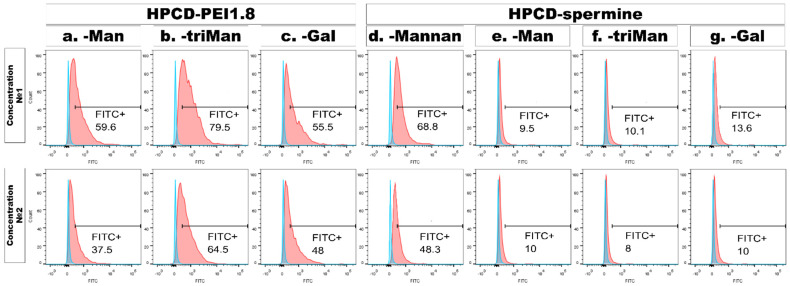
Flow cytometry results of macrophages incubated with polymer samples. Two types of polymers: HPCD-PEI1.8 and HPCD-spermine, modified with mannose (**a**,**e**), trimannose (**b**,**f**) or galactose (**c**,**g**) and one sample modified with mannan (**d**). Top row—concentration No. 1, bottom row—concentration No. 2 (two times less). Data are presented by overlaid histograms of control macrophages (blue) and macrophages after incubation with sample (red) represented by histograms. The percentage of cells in the gate is indicated under the name of the gate.

Seven days after adding PMA, we derived macrophage-like cells from the THP-1 cell line as were described before [104]. Most of these cells were CD206-positive according to immunocytochemical analysis (Figure 6 and Figure S10). CD206-positive cells effectively phagocytosed predominantly high-affinity polymeric conjugates with trimannoside or mannan: HPCD-PEI1.8-triMan, Mannan-spermine-HPCD, but not star-shaped CD, which was poorly non-specifically absorbed by macrophages. The capture of conjugates with linear mannose or galactose labels by macrophages occurs to a lesser extent and is nonspecific due to polymer adhesion (Figure 6 and Figure S10). A cytometry assay determined that 79.5% of macrophage-like cells were FITC-positive after adding HPCD-PEI1.8-triMan, 59.6% were FITC-positive after adding HPCD-PEI1.8-Man and 55.5% were FITC-positive after adding HPCD-PEI1.8-Gal (Figure 7) at concentration N^o^1. After decreasing the concentration of polymer twice, the uptake of FITC was 64.5%, 37.5% and 48.2%, respectively (Figure 7). Human dermal fibroblast showed a very low CD206 expression level and polymer uptake (the data is presented in our previous work [25]).

The data obtained on macrophages are consistent with the above-described primary model screening on the ConA model, which binds mannan and oligomannosides highly effectively, relates weakly to monosaccharides and very weakly to the negative control—galactose [17]. Moreover, we have previously carried out a computer modeling of the ConA and CD206 receptors: a similarity in ligand-binding patterns and mechanisms has been shown [22].

Interestingly, with HPCD-spermine-FITC conjugated with Man, triMan or Gal, the small particles were taken up by the cells significantly lower and showed no difference between labels and concentrations (Figure 6, Figure 7 and Figure S10). This group of ligands was specially synthesized with a negative surface charge due to COO^−^ groups left on cyclodextrin to clarify the role of the charge (zeta potential) of particles on the macrophage uptake. The FITC-positive macrophages in this group are approximately 10%, regardless of the affinity of the label on a highly charged particle. This indicates that the CD206+ dependent mechanism is realized only in the case of optimal structure and physico-chemical property (charge, mass) delivery systems.

Mannan grafted with HPCD was adsorbed by 68.8% and 43.1% of macrophage-like cells at concentrations 1 and 2, respectively (Figure 7). CD206+ macrophages preferentially phage large polymeric, lightly charged particles with a mass of at least 8 kDa, and small star-shaped cyclodextrins are not captured by them, even in the case of a high-frequency label.

Thus, it is shown that targeting macrophages due to the specific binding of large polymer particles with an oligo- or polymannose label with CD206 receptors is the basis for the implementation of platforms of enhanced forms of drugs.

### 2.5. Pharmacokinetics and Organ Distribution of MF in Molecular Containers

**Figure 8:**
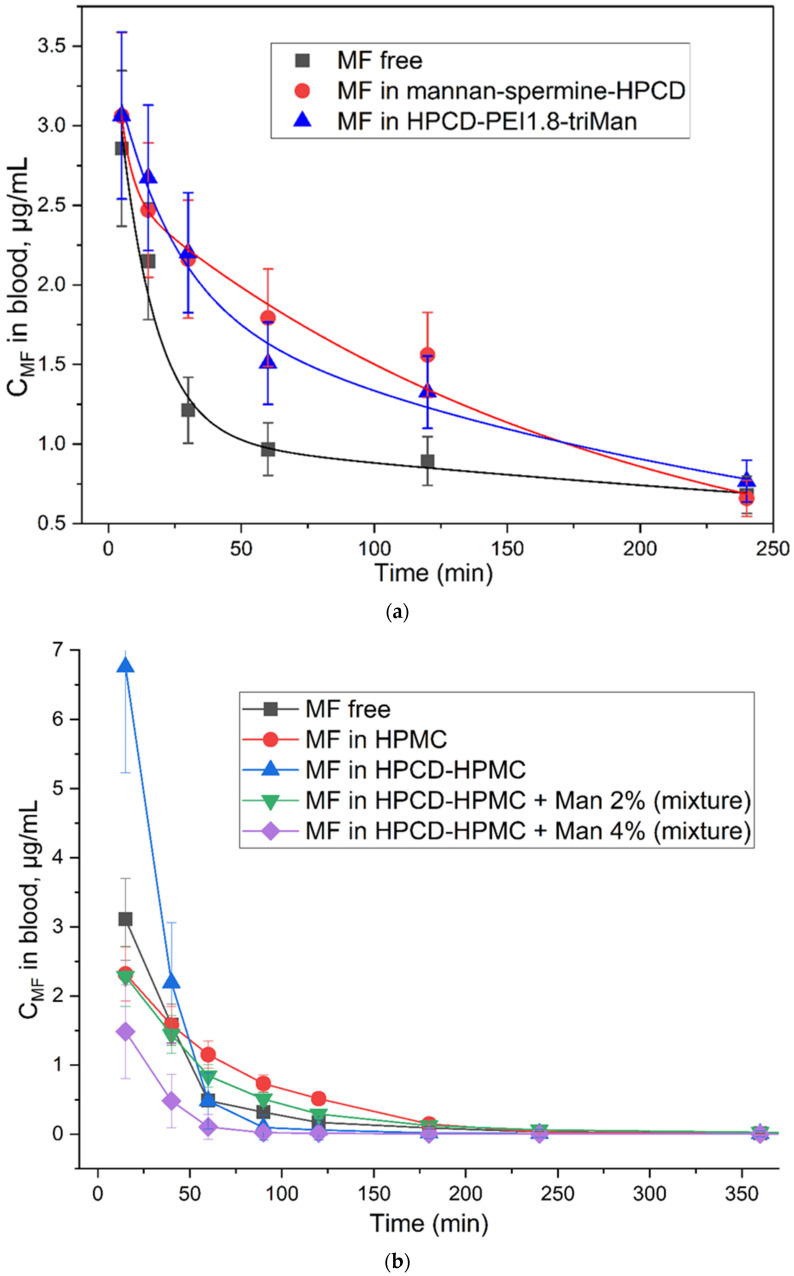
Pharmacokinetic curves: (**a**) MF and its formulations with mannosylated drug delivery systems in white mongrel rats; (**b**) MF and its formulations with HPMC in Wistar rats.

**Figure 9:**
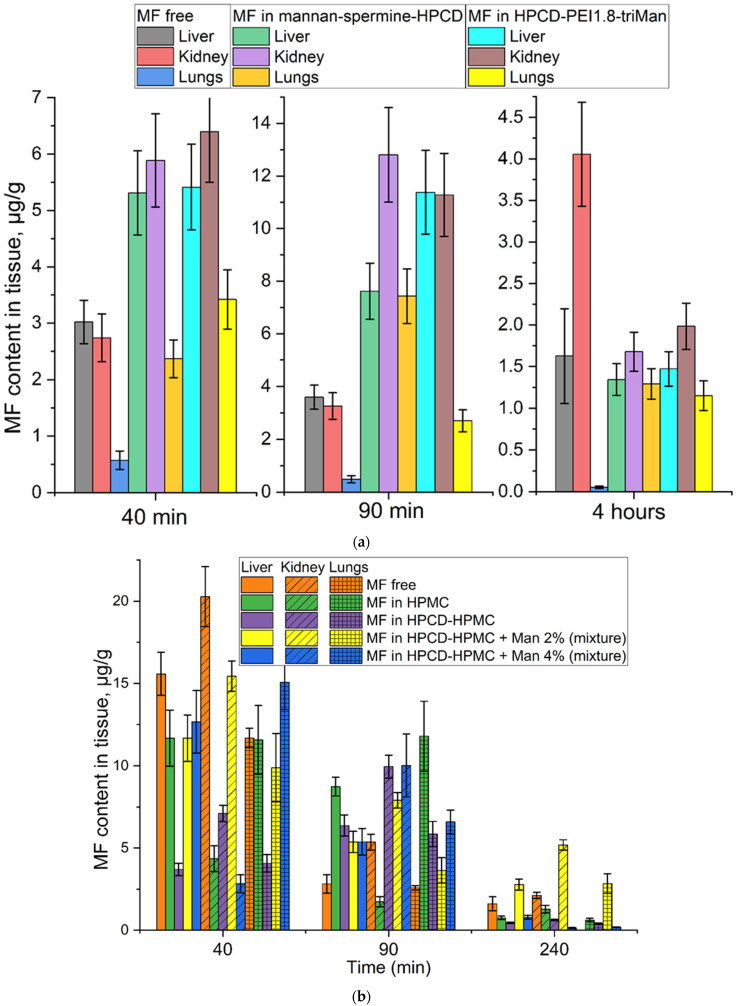
Biodistribution of: (**a**) MF and its formulations with mannosylated drug delivery systems in white mongrel rats; (**b**) MF and its formulations with HPMC in Wistar rats.

The main focus of this work is aimed at the development of molecular containers for drugs that will ensure the long-term and more effective action of antibacterial drugs, as well as their targeted delivery. Test samples: MF free and MF as part of delivery systems: Mannan-spermine-HPCD and HPCD-PEI1.8-triMan (Figure 8a and Figure 9a, Table 2). A series of carriers was selected as control samples: hydroxypropyl-methylcellulose (HPMC), its mixture with HPCD with a non-covalent mannose label (Figure 8b and Figure 9b, Table 3). These samples are necessary to explain the role of covalent multifunctional mannosylated systems in drug circulation in comparison with non-covalent mixtures. Mannan-spermine-HPCD and HPCD-PEI1.8-triMan significantly increase the MF circulation time and its efficiency (Figure 8a, Table 2). The half-life increases by 2–3 times due to a decrease in clearance and, as a result, the integral efficiency of AUC increases from 200 to 520–580 units. However, in the case of a simple HPMC, the effect is not so bright (Figure 8b, Table 3): the half-life increases by about 1.5 times, but the AUC increases slightly from 155 to 190 units. The addition of cyclodextrins to HPMC increases the loading of MF and causes the initial accelerated excretion of the drug with subsequent prolonged effect. However, these mixtures are ineffective, and their use is unjustified. The role of the covalent mannose label (high affinity to CD206 trimannoside) in the composition of the developed systems is proved by the fact that the introduction of mannose into the HPCD-HPMC mixture worsens the characteristics due to the loosening of the carrier structure (Table 3). Thus, it is the polymers grafted with cyclodextrins that have a significant potential for enhancing antibiotics and improving their pharmacokinetic parameters.

An important parameter is organ bioavailability and distribution (Figure 9). Mannan-spermine-HPCD and HPCD-PEI1.8-triMan double or triple the accumulation of MF in the liver and kidneys during the first one or two hours, while the antibiotic content in the lungs increases 5–10 times and is maintained even after 4 h at the level of 1.2–1.4 μg/g of tissue. In comparison with the free MF, this is a fantastic effect. Non-covalent mixtures (Figure 9b) in the first 40 min do not significantly change the accumulation of MF in the lungs, while after 90 min, the effect becomes bright and lasts up to 4 h. However, the only sample that demonstrated effectivity was HPCD-HPMC with 2% noncovalent mannose. Although, conjugated systems are much more efficient.

## 3. Materials and Methods

### 3.1. Reagents

Carbonyldiimidazole (CDI) was obtained from GL Biochem Ltd. (Shanghai, China) via an intermediary Himprocess (Moscow, Russia). ConA was purchased from Paneco-ltd (Moscow, Russia). Mannan (46 kDa), D-mannose, PEI 1.8 kDa and PEI 10 kDa (branched), fluorescein isothiocyanate (FITC), EDTA, DMF, DMSO, Et_3_N, 2-hydroxypropyl-β-cyclodextrin (HPCD), 1M 2,4,6-trinitrobenzenesulfonic acid and moxifloxacin hydrochloride were obtained from Sigma Aldrich (St. Louis, MI, USA). Mono-(6-(1,6-hexamethylenediamine)-6-deoxy)-β-cyclodextrin (amCD) was supplied by Dayang Chem Co., Ltd. (Hangzhou, China). Mannotriose-di-(N-acetyl-D-glucosamine) was obtained from Dayang Chem Co., Ltd. (Hangzhou, China). Menthol was purchased from Rotichrom GC (Carl Roth GmbH + Co. Karlsruhe, Germany). Eugenol and safrole at the highest commercial quality were purchased from Acros Organics (Flanders, Belgium). Other chemicals: NaBH_4_, salts and acids were from Reakhim Production (Moscow, Russia).

### 3.2. Synthesis and Characterization of Mannosylated Conjugates

Activated HPCD was synthesized as described earlier [70]. Briefly, the slow drop-by-drop addition of HPCD solution in water (0.4 mM) to the CDI solution in DMSO (1.5 mM) led to the formation of HPCD-CDI_7_ composition at the output. 

Ligands 1a, 1b (Figure 1a). A 50 mg mannan sample was dissolved in 5 mL of 1 mM HCl, then a 0.2 molar excess of KJO_4_ relative to mannose units was added. The mixture was incubated for 30 min at 40 °C. Periodate purification was performed using dialysis (cut-off 6–8 kDa) against water for 2 h. We brought the pH of the system to 9–10. In the case of ligand 1a, an equimolar amount of amCD was added. In the case of 1b, spermine and the mixtures were incubated for an hour at 40 °C. The reduction of Schiff bases was carried out using sodium borohydride. Additionally, a 1.5-fold molar excess of activated HPCD relative to spermine was added to system 1b, and incubation was continued at the same temperature for an hour. The final purification of 1a, 1b samples was performed using 24 h dialysis (cut-off 6–8 kDa).

Ligands of the second series 2a, 2b, 2c (Figure 1b). Introduction of the FITC label (used for the synthesis of conjugates for experiments with macrophages). To the aqueous solution of PEI1.8 (5% in 0.01M HCl, 1 g), a solution of FITC (15 mg in 1.5 mL DMSO) was added drop by drop with stirring; the pH was brought to 10. The mixture was incubated at 40 °C for 1 h, followed by purification by dialysis against water (cut-off 1 kDa) for 6 h. Then, to the PEI1.8-FITC solution (or simply PEI1.8), HPCD-CDI_7_ was added based on an excess of HPCD over PEI 5 to 1. The mixture was incubated at 40 °C for 2 h at pH 9 with subsequent purification by dialysis against water (3 kDa) for 6 h. The sample was divided into three equal parts, to which an aqueous solution of 50 mg/mL of mannose (20 times molar excess over PEI)—2a, triMan (6 times molar excess over PEI)—2b and galactose (20 times molar excess over PEI)—2c were added, respectively. The number of pyranose residue is unified for the relevance of affinity comparison and experiments with macrophages. The mixtures were incubated for a day at pH 10 and 40 °C followed by the addition of 1.5-molar excess of NaBH_4_ relative to Man, Gal or triMan, and 6 h incubation with subsequent purification by dialysis (cut-off 3 kDa) for 6 h.

Ligands of the third series 3a, 3b, 3c (Figure 1c). HPCD-CDI_7_ in 50% DMSO was added drop by drop to the 15-molar excess spermine solution (20 mg/mL, pH 10), followed by 2 h incubation at 40 °C and purification by dialysis against water (cut-off 1 kDa) for 6 h. Then, a two-fold molar excess of FITC (10 mg/mL in DMSO) relative to HPCD was added to the HPCD-spermine_4_ solution (experimentally—3–5 molecules of spermine account for one round of HPCD) drop by drop with stirring. The pH was brought to 10. Further, the synthesis was carried out similarly to the technique for ligands of the 2nd series with the presence of an excess of sugars at the last stage: nine for Man and Gal, three for triMan.

The purification of the samples was performed by HPLC gel filtration in a Knauer chromatography system (Knauer, Berlin, Germany) on Diasfer-110-C18 column (BioChemMack, Moscow, Russia): grains—6 μm, size 4 × 150 mm. The eluent was CH_3_CN-H_2_O (90:10, *v*:*v*); the elution rate was 0.8 mL/min, 25 °C. Due to the high polydispersity of the conjugates, fractions corresponding to maxima ± 0.5 min by retention time were used in the experiments. The chromatogram of the resulting conjugates is shown in Supplemental Materials (Figure S5).

All samples were freeze-dried for two days at –60 °C (Edwards 5, BOC Edwards, UK). The degree of mannosylation was calculated according to spectrophotometric titration of amino groups (before and after mannosylation) with 2,4,6-trinitrobenzenesulfonic acid [70,86]. Mainly, primary amino groups are detected.

Determination of the hydrodynamic diameter of the synthesized polymers was carried out by NTA (Nanoparticle Tracking Analysis) using a Nanosight LM10-HS device (Great Britain). Samples were diluted with MilliQ purified water to a concentration of 10^7^—10^9^ particles/mL and kept in an ultrasonic bath for 30 s. The hydrodynamic diameter was determined by the Stokes–Einstein equation due to the analysis of the trajectory of Brownian motion of particles. Each sample was measured three times. The hydrodynamic diameter of the particles was also determined using the method of dynamic light scattering.

### 3.3. FTIR Spectroscopy

ATR-FTIR spectra of samples’ solutions were recorded using a Bruker Tensor 27 spectrometer equipped with a liquid nitrogen-cooled MCT (mercury cadmium telluride) detector. Samples were placed in a thermostatic cell BioATR-II with ZnSe ATR element (Bruker, Bremen, Germany). The FTIR spectrometer was purged with a constant flow of dry air (Jun-Air, Michigan, USA). FTIR spectra were acquired from 900 to 3000 cm^−1^ with 1 cm^−1^ spectral resolution. For each spectrum, 50–70 scans were accumulated at 20 kHz scanning speed and averaged. Spectral data were processed using the Bruker software system Opus 8.2.28 (Bruker, Bremen, Germany), which includes linear blank subtraction, baseline correction, differentiation (second order, 9 smoothing points), min-max normalization and atmosphere compensation [70,105]. If necessary, 11-point Savitsky–Golay smoothing was used to remove noise. Peaks were identified by standard Bruker picking-peak procedure.

### 3.4. NMR Spectroscopy

An amount of 12 mg of the sample was dissolved in 700 μL of D_2_O. ^1^H NMR spectra of the solutions were recorded on a Bruker Avance 400 spectrometer (Bremen, Germany) with an operating frequency of 400 MHz. Chemical shifts are given in ppm on the δ scale relative to hexamethyldisiloxane as an internal standard. The analysis and processing of NMR spectra were performed in the program MestReNova v. 12.0.0-20080.

### 3.5. Dynamic Light Scattering (DLS)

The particle sizes and zeta potentials were measured using a Zetasizer Nano S «Malvern» (Worcestershire, UK) (4 mW He–Ne-laser, 633 nm, scattering angle 173°). The experiment was performed in a temperature-controlled cell at 25 °C. Autocorrelation functions of intensity fluctuations of light scattering were obtained using the correlation of the Correlator system K7032-09 «Malvern» (Worcestershire, UK). Experimental data were processed using «Zetasizer Software» (v. 8.02).

### 3.6. Macrophages Study

#### 3.6.1. Cell Lines and Modelling

For the macrophage phagocytose assay, a human monocyte cell line THP-1 was used. Cells were obtained from the bank of cell lines of Lomonosov Moscow State University. THP-1 cells were cultured on RPMI-1640 (Gibco, Carlsbad, CA, USA), supplemented with GlutaMAX™ Supplement (Gibco, Carlsbad, CA, USA) and buffered with 10 mM HEPES pH 7.4 containing 10% heat-inactivated FBS (Gibco, Carlsbad, CA, USA) and 1% antimycotic antibiotic (HyClone) at 37 °C and 5% CO_2_. Macrophage-like cells were derived by adding to THP-1 (0.5 million cells/mL) 100 nM phorbol 12-myristate 13-acetate (PMA, p8139, Sigma Aldrich, St. Louis, MI, USA) for 72 h. After 72 h, the medium was replaced and cells were cultured for another 96 h.

Human dermal fibroblasts were isolated from skin obtained from healthy donors during abdominal surgery. All donors, or if donors were under 18, a parent and/or legal guardian, gave their informed consent and the local ethics committee of the Medical Research and Education Center of Lomonosov Moscow State University (IRB00010587, Moscow, Russia) approved the study protocol (#4, 4 June 2018). Cells were cultured on DMEM with low glucose (Gibco) supplemented with GlutaMAX™ Supplement (Gibco, CA, USA) containing 10% FBS (Gibco) and 1% antimycotic antibiotic (HyClone) at 37 °C and 5% CO_2_.

#### 3.6.2. Phagocytosis Assay

Dry samples of: 2a-2c–1.8 kDa polyethylenimines with grafted CD; 1b—mannan with grafted CD; 3a-3c—star-shaped CD with spermines (Table S1) conjugated with a FITC fluorescent label were diluted in PBS (PanEco) with 10% DMSO (Sigma-Aldrich, St. Louis, MI, USA). Macrophage-like cells were washed with Hanks’ solution (PanEco) and polymers were added at final concentration №1 according to Table S1 in serum-free media. Concentration №2 was obtained by diluting the first sample twice. As a control, we used macrophage-like cells without adding polymer. As a control of specific CD206-mediated binding, we used human dermal fibroblasts, which did not express CD206. For cytometry assay, cells were incubated for 40 min, washed thrice with Hank’s solution and scraped in Versen’s solution. Cell suspension was centrifuged at 300× *g* for 5 min at +4 °C. The cell pellet was resuspended in a PBS solution. For immunocytochemistry assay, cells were incubated for 4 min, washed with PBS and fixed with 4% formaldehyde solutions (Panreac).

#### 3.6.3. Cytometry

Cytometry was performed on a BD FACSAria™ III Cell Sorter instrument. The fluorescence of the FITC-conjugated polymer captured by the cells was analyzed (Figure S11). There was a change in the percentage of cells fluorescing in the FITC channel. Cells not incubated in the sample solution were evaluated (Control).

#### 3.6.4. Immunocytochemistry

To block nonspecific binding sites, cells were incubated with a 10% solution of normal goat serum (Sigma, St. Louis, MI, USA) in PBS with the 1% bovine serum albumin BSA (PanEco) for 1 h at RT. Then, the samples were incubated with a solution of anti-CD206 antibodies (ab64693, Abcam, 1:100) or rabbit polyclonal control IgG (910801, Biolegend) as a control for 2 h at RT, and subsequently with Goat anti-Rabbit antibody conjugated with Alexa594 (A11037, Invitrogen, 1:1000). The nuclei were labeled with DAPI (Sigma, St. Louis, MI, USA). Samples were analyzed with a Leica DM6000B fluorescent microscope equipped with a Leica DFC 360FX camera (Leica Microsystems GmbH, Wetzlar, Germany).

### 3.7. In Vivo Experiments

#### 3.7.1. Animals

The experiments were carried out on Wistar rats weighing (400 ± 20) g and white mongrel rats weighing (300 ± 20) g kept under a 12-h light regime and a standard diet. Before the experiment, rats were anesthetized and two catheters were implanted: one into a jugular vein for drug administration, the second into the carotid artery for blood sampling.

#### 3.7.2. Protocol of Experiments on the Study of MF Pharmacokinetics and MF Accumulation in Organs

The drugs were administered intravenously to animals by 1 mL into the tail vein at the rate of 5 mg/mL per MF. The dose of the administered drug according to MF was 15 mg/kg, which corresponds to a human dose of 2.5 mg/kg (150–250 mg depending on the weight of the subject). Blood sampling was carried out in tubes with K-EDTA (Sarstedt, Germany) from the pineal vein after 5, 15, 40, 60 min, 2 h and 4 h (after 4 h, the MF content in the blood was minimal). After 40, 90 and 240 min following intravenous administration of the drugs, the animals were slightly anesthetized; after decapitation, the liver, kidneys and lungs were taken for analysis. After appropriate sample preparation, a quantitative analysis of MF content in blood and organs was performed using fluorescence spectroscopy (λ_exci_ = 290 nm, emission in range 400–500 nm with maximum at 460 nm). Registration of fluorescence spectra was carried out using a SpectraMax M5 device (Pennsylvania, USA) in the Costar black\clear bottom tablet (96 wells). T = 25 °C.

#### 3.7.3. Sample Preparation of Blood and Organ Samples

An amount of 450 µL of cold methanol was added to 50 µL of blood with constant stirring. Extracts were centrifuged for 10 min at 10,000× *g* and a temperature of 4 °C for protein precipitation on an Eppendorf 5417R centrifuge. An amount of 300 µL of water was added to the tissue suspension (100 ± 5 mg) and homogenized using Sonopuls HD 2070/2200 ultrasonic homogenizer with MS-72 tip (Bandelin, Germany) at minimum power. Then, 450 µL of cold methanol was added drop by drop to 50 µL of homogenate. After intensive mixing for 3–5 min, the precipitation of proteins was carried out by centrifugation under the same conditions. The supernatant was analyzed for the content of MF.

### 3.8. Mathematical Calculations and Equations

(1) Calculation of the dissociation constants ConA–ligand. Consider the equilibrium: ConA + n ligand ↔ ConA·nligand, where *K*_d_ = [ligand]^n^ · [ConA]/[ConA·n ligand].

First, the fitting of the curves of change of the analytical signal ξ (peak intensity or peak position in FTIR native or second-derivative spectra) versus the concentration of the ligand was carried out using the Hill equations: (1) ξ = ξ_∞_ · [ligand]^n^/([ligand]^n^ + *K*^n^), where ξ_∞_—horizontal asymptote; (2) ξ = ξ_0_ + (ξ_∞_ − ξ_0_) · [ligand]^n^/([ligand]^n^ + *K*^n^). Second, calculation n and *K*_d_ values by Hill’s linearization in n-binding site model: lg (θ/(1 − θ)) = n · lg [L] − lg *K*_d_, where θ = | (ξ − ξ_0_)/(ξ_∞_ − ξ_0_) |, is a fraction of the bound substance. n in Hill equation–number of ligand molecules per one ConA subunit.

(2) The number of loaded MF molecules and adjuvants EG, safrole and menthol in molecular containers was calculated by subtracting from the FTIR spectrum of the complex spectra of each component until the characteristic peaks were aligned: menthol—2800–3000 cm^−1^, EG—1515 cm^−1^, safrole—1502 and 1487 cm^−1^, and finally MF (since the peak of MF overlaps, it was subtracted last)—1440 cm^−1^.

(3) Calculations of pharmacokinetic parameters were performed using the Borgia program (v. 1.03, NPP “Nauka Plus”). Data for MF and its formulations with HPMC on awake Wistar rats were analyzed using a single-compartment model without absorption: C_MF_(t) = C_MF_(0)*exp(–k*t). Half-elimination period τ_1/2_ = ln2/k; distribution volume V_d_ = dose/C_MF_(0), where dose = 5 mg; clearance Cl = k*V_d_.

Data for MF and its formulations with mannosylated drug delivery systems were analyzed using a two-compartment model without absorption: C_MF_(t) = A*exp(–k_12_*t) + B*exp(–k_21_*t), where k_12_ and k_21_—kinetic constants, A and B are concentration constants and A + B = C_MF_(0).

(4) Statistical analysis of obtained data was carried out using the Student’s t-test Origin 2022 software (OriginLab Corporation). Values are presented as the mean ± SD of three experiments.

## 4. Conclusions

This paper presents experimental evidence of the effectiveness of using targeted drug delivery systems to macrophage mannose receptors to create new improved forms of therapeutic forms of drugs. The focus of attention is directed to the problem of resistant and dormant pathogens localized in macrophages. Access to the sites of localization of pathogens is provided by polymer mannosylated systems that circulate in the body much longer and penetrate more into the lungs—to alveolar macrophages. The specificity of delivery systems to CD206+ macrophages was visualized using an immunocytochemical experiment: large polymers with a trimannoside label or mannan in their composition are most effectively captured by macrophages. At the same time, conjugates with linear mannose and galactose labels are absorbed less specifically—what correlates with the data of primary screening on the ConA receptor model—which turned out to be a relevant model of the CD206 macrophage mannose receptor. In vivo experiments have shown an improvement in the pharmacokinetic parameters of moxifloxacin in the composition of polymer molecular containers in comparison with the simple form by several times: clearance was reduced to three times; the time of active action was increased up to 10 times; bio-distribution to the lungs in the first hours after administration of the drug was noticeably greater and after 4 h of observation, free moxifloxacin was practically removed from the lungs of rats, while in polymer systems its content was significant—1.2 µg/g. Therefore, the conducted research opens up new opportunities to influence complex macrophage-associated infections. The data obtained in combination with previous studies form the basis for choosing the optimal delivery system and adjuvant for a specific therapy task and possibly a specific patient.

## Figures and Tables

**Table 1:** Characteristics of drug delivery systems and their affinity to mannose receptor.

Ligand Designation	Carrier *	Label (*n*) **	Molecular Weight, kDa	Hydrodynamic Size ***, nm	Zeta-Potential ****, mV	Polydispersity Index	−lg K_d_ (ConA–Ligand)
**1a**	Mannan-amCD-FITC (1:15:3)	Mannan itself	70 ± 12	140 ± 40	3.5 ± 2.1	0.5	6.3 ± 0.3
**1b**	Mannan-spermine-HPCD-FITC (1:15:10:2)		65 ± 11	145 ± 50	2.5 ± 3.3	0.6	6.8 ± 0.3
**2a**	HPCD-PEI1.8-FITC * (3:1:0.5:n) **	Man (12)	9 ± 3	176 ± 100	–4 ± 2	0.4	5.3 ± 0.4
**2b**		triMan (3)		120 ± 50	–6.5 ± 1.5	0.45	6.6 ± 0.4
**2c**		Gal (12)		170 ± 60	–7 ± 3	0.4	3.0 ± 0.2
**3a**	HPCD-spermine-FITC (1:4:0.5:n) **	Man (3)	3 ± 1	70 ± 25	–27 ± 5	0.2	5.4 ± 0.3
**3b**		triMan (1–2)		80 ± 30	2 ± 9	0.3	6.0 ± 0.3
**3c**		Gal (3)		65 ± 20	–23 ± 5	0.2	3.3 ± 0.4

* FITC-labeled ligands were used only for experiments with macrophages. ** n is the number of carbohydrate labels. *** by NTA. **** by DLS.

**Table 2:** Pharmacokinetic parameters of MF in white mongrel rats. Drugged animals. The study time is 4 h. Two-compartment model without absorption.

MF Form	Half-Distribution Period, Min	Half-Elimination Period, Min	Distribution Volume in Central Camera, L	Kinetical Distribution Volume, L	Stationary Distribution Volume, L	Clirens, mL/min	Area under Curve (AUC), μg·min/mL	Mean Residence Time, min
MF free	11 ± 1	85 ± 5	1.4 ± 0.1	3.1 ± 0.2	2.6 ± 0.1	25.5 ± 0.5	200 ± 15	100 ± 15
MF in mannan-spermine-HPCD	4.6 ± 0.5	140 ± 15	1.3 ± 0.1	2.0 ± 0.2	1.9 ± 0.2	9.7 ± 1.1	520 ± 60	200 ± 25
MF in HPCD-PEI1.8-triMan	19 ± 2	210 ± 20	1.5 ± 0.1	2.6 ± 0.2	2.4 ± 0.2	8.6 ± 1.0	580 ± 70	280 ± 30

**Table 3:** Pharmacokinetic parameters of MF in Wistar rats. Awake animals, the study time is 48 h. Single-compartment model without absorption.

MF Form		Half-Elimination Period τ_1/2_, min	Elimination Constanta k, min^−1^	Distribution Volume V_d_, L	Clirens Cl, mL/min	Area under Curve (AUC), μg·min/mL
MF free		26 ± 2	0.027 ± 0.002	1.2 ± 0.1	32 ± 2	155 ± 5
HPMC		40 ± 4	0.017 ± 0.002	1.6 ± 0.1	27 ± 2	190 ± 10
HPCD-HPMC	0% Man—noncovalent	14 ± 1	0.049 ± 0.004	0.35 ± 0.05	17 ± 1	290 ± 20
	2% Man—noncovalent	34 ± 2	0.020 ± 0.002	1.6 ± 0.1	32 ± 2	155 ± 5
	4% Man—noncovalent	14 ± 1	0.048 ± 0.003	1.6 ± 0.1	78 ± 6	64 ± 4

## Data Availability

The data presented in this study are available in the main text and Supplementary Materials.

## Supplementary Materials

The following supporting information can be downloaded at: https://www.mdpi.com/article/10.3390/ijms232416144/s1.

## Abbreviations

amCD—mono-(6-(1,6-hexamethylenediamine)-6-deoxy)-β-cyclodextrin; CD—cyclodextrin; EE—entrapment efficiency; EG—eugenol; FITC—fluorescein isothiocyanate; HPCD—(2-hydroxypropyl)-β-cyclodextrin; HPMC—hydroxypropyl methylcellulose; LC—loading capacity; MF—moxifloxacin; PEI—polyethyleneimine; triMan—mannotriose residue.
