# Supplementary material for: Mannosylated Systems for Targeted Delivery of Antibacterial Drugs to Activated Macrophages
Source: Int J Mol Sci. 2022 Dec 18;23(24):16144. doi: 10.3390/ijms232416144 (PMC9787453; doi:10.3390/ijms232416144)
Supplement: Supplementary file 1 [file ijms-23-16144-s001.zip › ijms-2038634-supplementary.pdf]

Supplementary Materials

Mannosylated systems for targeted delivery of antibacterial drugs to activated macrophages

Igor D. Zlotnikov [1,*], Maksim A. Vigovskiy [2,3], Maria P. Davydova [3], Milan R. Danilov [1], Ulyana D. Dyachkova [2,3], Olga A. Grigorieva [2,3] and Elena V. Kudryashova [1,*]

[1] Faculty of Chemistry, Lomonosov Moscow State University, Leninskie Gory, 1/3, 119991 Moscow, Russia

[2] Institute for Regenerative Medicine, Medical Research and Education Center, Lomonosov Moscow State University, 27/10, Lomonosovsky Ave., 119192 Moscow, Russia

[3] Faculty of Medicine, Lomonosov Moscow State University, 27/1, Lomonosovsky Ave., 119192 Moscow, Russia

* Correspondence: zlotnikovid@my.msu.ru (I.D.Z.); helenakoudriachova@yandex.ru (E.V.K.)

Content

Figure S1. Fourier transform infrared spectra and scheme of synthesis of mannan and periodate-oxidized mannan (mannan-oxi). Aqueous solutions. T = 22°C.

Figure S2. Fourier transform infrared spectra of HPCD-PEI1.8-FITC with grafted Man or Gal or triMan. T = 22 °C.

Figure S3. Particle size distribution by NTA method: (a) HPCD-PEI1.8-triMan, (b) Mannan-spermine-HPCD, (c) HPCD-spermine-triMan. PBS (pH = 7.4). T = 25 °C.

Figure S4. Particle zeta-potential distribution by DLS method: (a) Mannan-spermine-HPCD, (b) HPCD- spermine-triMan, (c) HPCD- spermine-Man, (d) HPCD-spermine-Gal. PBS (pH = 7.4). T = 25 °C.

Figure S5. High performance liquid chromatogram of mannosylated drug delivery systems. Knauer chromatography system (Knauer, Berlin, Germany) on Diasfer-110-C18 column (BioChemMack, Moscow, Russia): grains – 6 µm, size 4×150 mm. The eluent was CH_3CN-H_2O (90:10, v:v); the elution rate was 0.8 mL/min, 25 °C.

Figure S6. Dependence of the change in the intensity of the absorption band of Amide II in the FTIR spectra of ConA when binding to ligands. T = 22 °C.

Figure S7. Amide I peak deconvolution of (a) ConA and (b) ConA complex with mannan-amCD FTIR spectra. (c) Dependences of the proportions of components in the secondary structure of ConA during ligand (mannan-amCD) binding. (d) Changes in the aggregation of ConA when binding to a ligand (mannan-amCD) relative to the native form. C_0(ConA subunit) = 0.1 mM. T = 22 °C.

Figure S8. Fourier transform infrared spectra of **(a)** EG (eugenol) alone and its complexes with PEI1.8-triMan; **(b)** Safrole alone and its complexes with PEI1.8-triMan; **(c)** FTIR spectra – loading of four components into a molecular container at once: moxifloxacin 0.9 mM, eugenol 17 mM, safrole 12 mM, menthol 12 mM. T = 22 °C.

Figure S9. ^{1}H NMR spectra of **(a)** MF, **(b)** MF complexed with mannan-spermine-HPCD (w:w = 1:1). Proton assignment is given. D_2O, 400 MHz.

Figure S10. Immunocytochemical evaluation of CD206 in THP-1 derived macrophage-like cells (red channel). Phagocytosis assay with polymers (according to table S1) at concentration №2 conjugated with a FITC fluorescent label after incubation for 40 min (green channel). Phase contrast microscopy, fluorescent microscopy, blue channel —nuclei stained with DAPI. Scale bar, 100 µm.

Figure S11. Flow cytometry of macrophage, gating strategy. (A) – exclusion of cellular debris based on forward and side light scattering, (B) – selection of single cells by forward scatter signal area and height, (C) – selection of single cells by side scatter signal width and height. (D,E) – overlay of a control sample (blue) and a sample after incubation with one of the polymers (red) represented by a dot plot (D) and a histogram (E). The percentage of cells in the gate is indicated under the name of the gate.

Table S1. Characteristics of polymer conjugates used for experiments with macrophages: capture efficiency, specificity – depending on the molecular architecture, ligand components and carbohydrate label.

Figure S1. Fourier transform infrared spectra and scheme of synthesis of mannan and periodate-oxidized mannan (mannan-oxi). Aqueous solutions. T = 22°C.

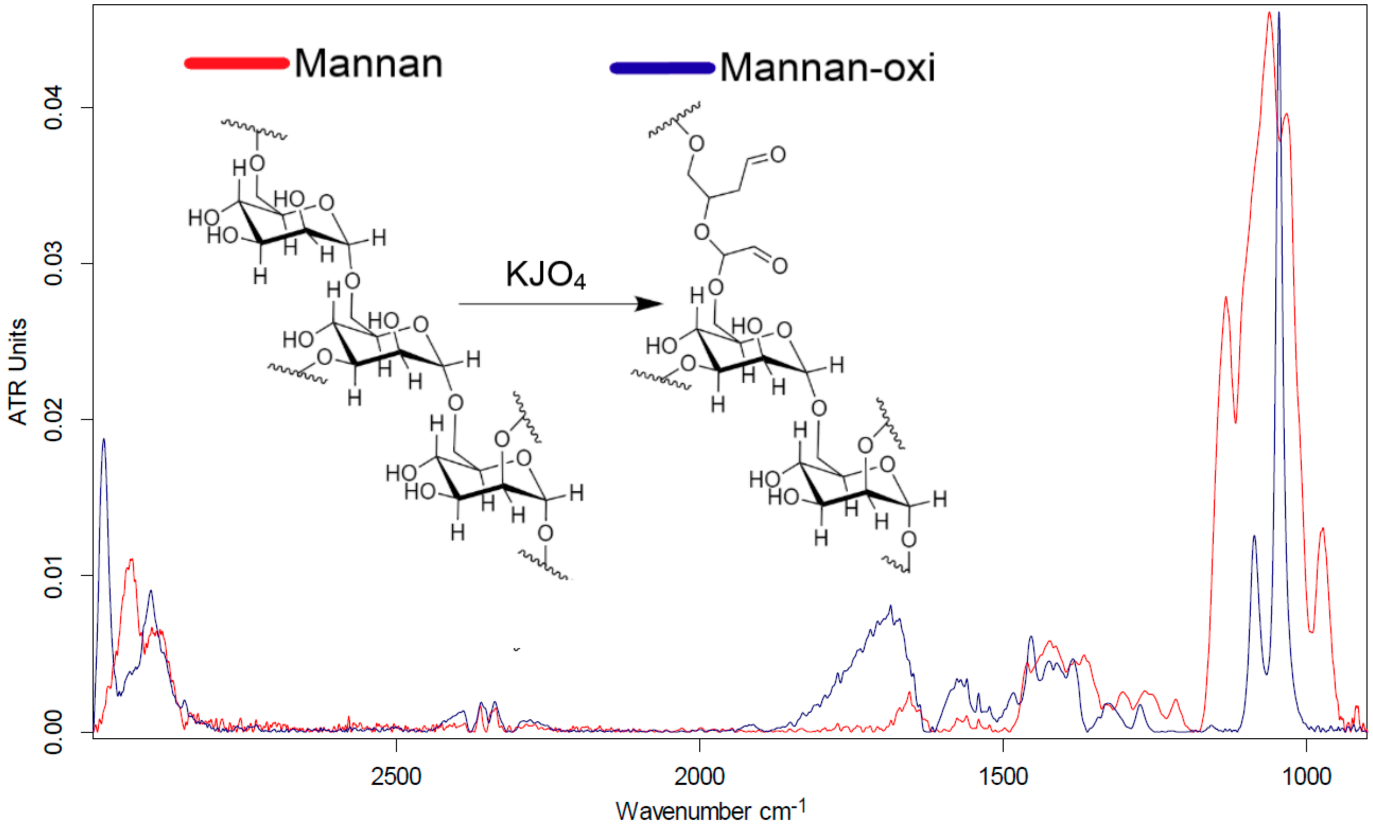

Figure S2. Fourier transform infrared spectra of HPCD-PEI1.8-FITC with grafted Man or Gal or triMan. T = 22 °C.

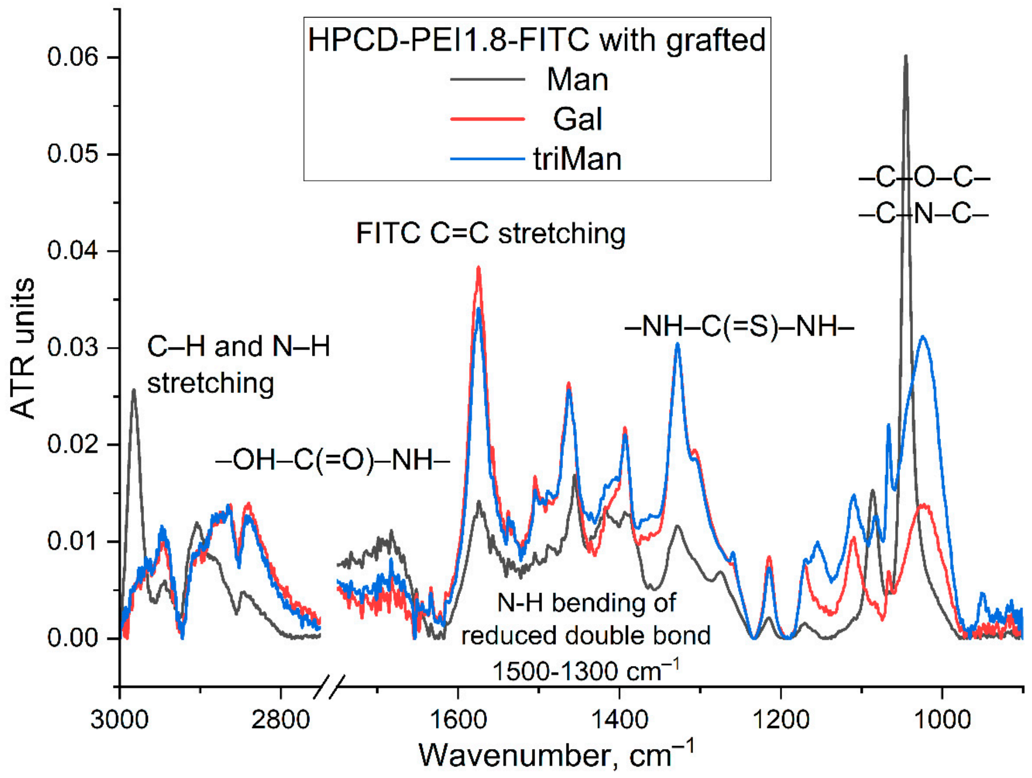

(a)

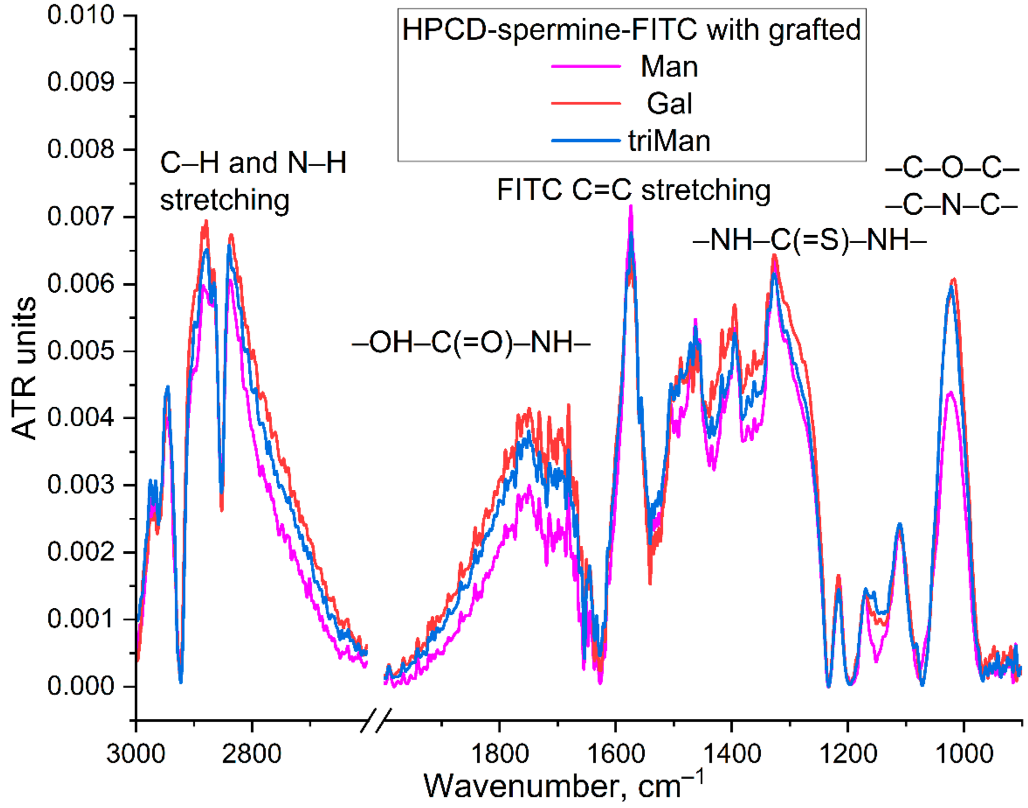

HPCD-spermine-FITC with grafted
Man
Gal
triMan
C–H and N–H
stretching
FITC C=C stretching
–C–O–C–
–C–N–C–
–NH–C(=S)–NH–
–OH–C(=O)–NH–
ATR units
0.010
0.009
0.008
0.007
0.006
0.005
0.004
0.003
0.002
0.001
0.000
3000
2800
1800
1600
1400
1200
1000
Wavenumber, cm–1

(b)

Figure S3. Particle size distribution by NTA method: (a) HPCD-PEI1.8-triMan, (b) Mannan-spermine-HPCD, (c) HPCD-spermine-triMan. PBS (pH = 7.4). T = 25 °C.

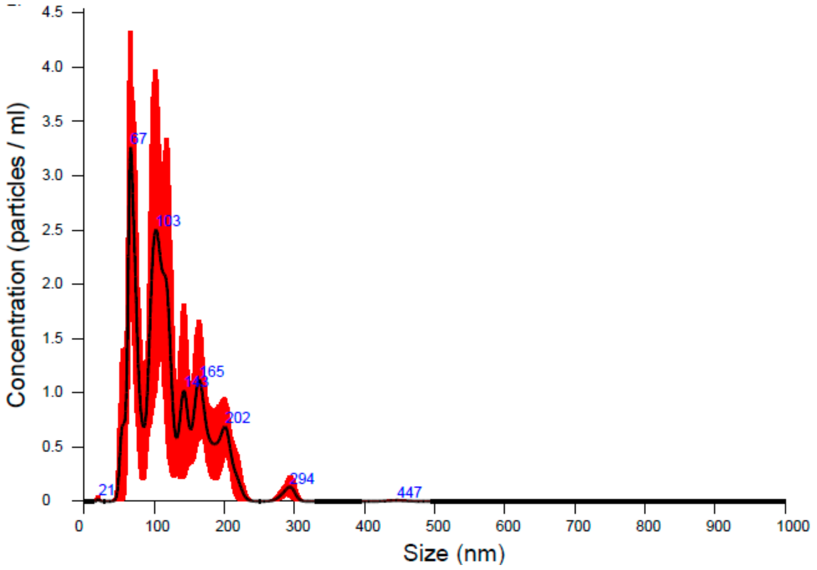

(a)

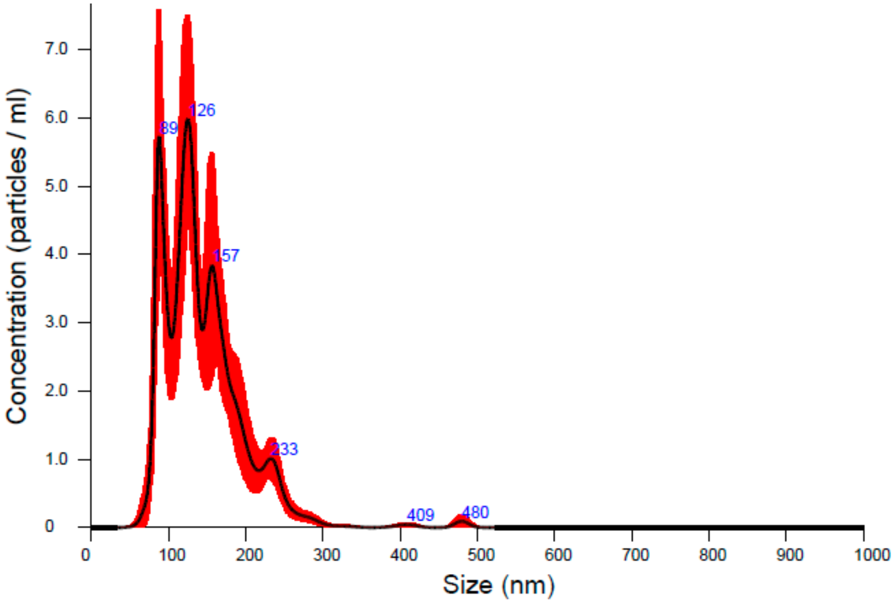

Concentration (particles / ml)
7.0
6.0
5.0
4.0
3.0
2.0
1.0
0
89
126
157
233
409
480
0
100
200
300
400
500
600
700
800
900
1000
Size (nm)

(b)

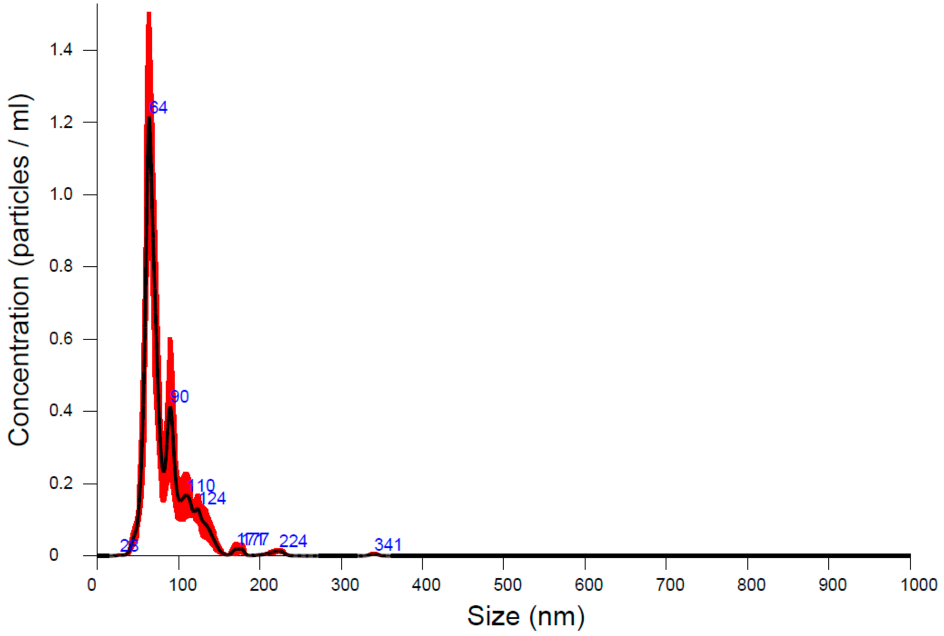
Concentration (particles / ml)
Size (nm)
1.4
1.2
1.0
0.8
0.6
0.4
0.2
0
0
100
200
300
400
500
600
700
800
900
1000
64
90
110
124
224
341

(c)

Figure S4. Particle zeta-potential distribution by DLS method: (a) Mannan-spermine-HPCD, (b) HPCD- spermine-triMan, (c) HPCD- spermine-Man, (d) HPCD-spermine-Gal. PBS (pH = 7.4). T = 25 °C.

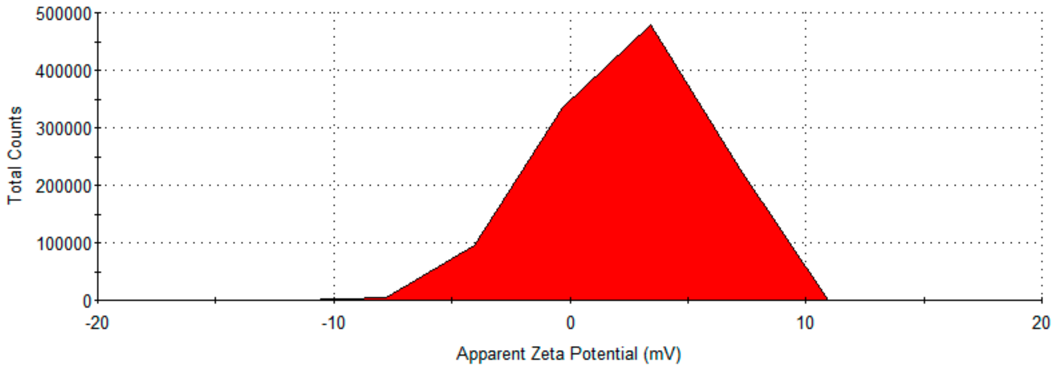

(a)

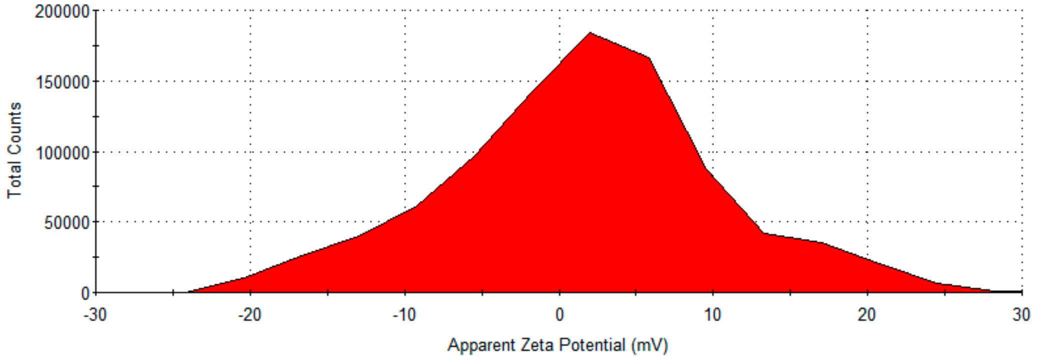

(b)

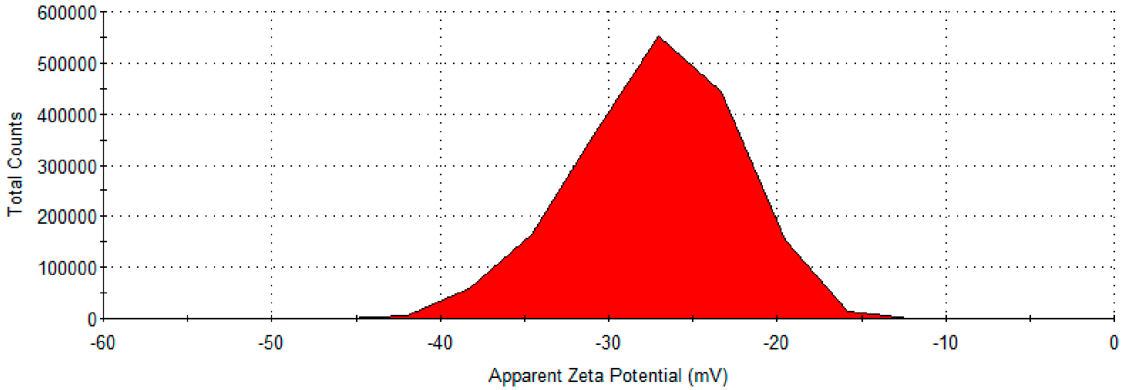
600000
500000
400000
300000
200000
100000
0
Total Counts
-60
-50
-40
-30
-20
-10
0
Apparent Zeta Potential (mV)

(c)

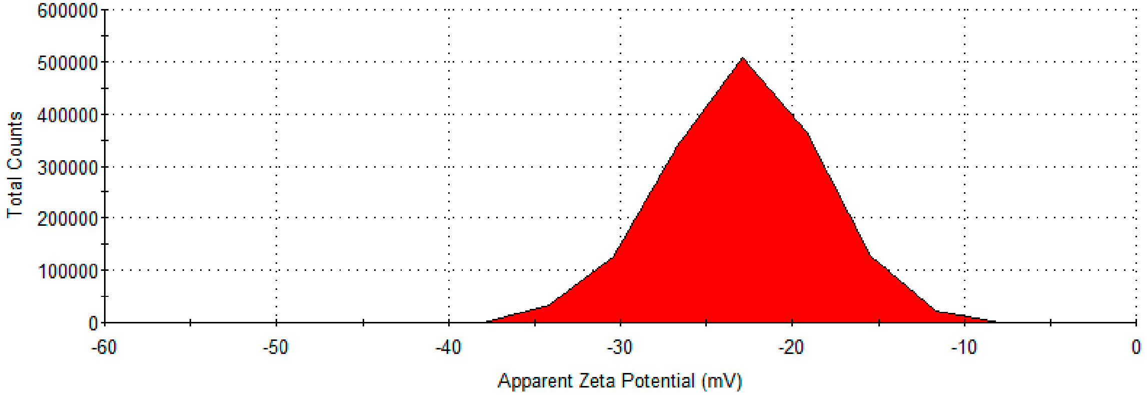
600000
500000
400000
300000
200000
100000
0
Total Counts
-60
-50
-40
-30
-20
-10
0
Apparent Zeta Potential (mV)

(d)

Figure S5. High performance liquid chromatogram of mannosylated drug delivery systems. Knauer chromatography system (Knauer, Berlin, Germany) on Diasfer-110-C18 column (BioChemMack, Moscow, Russia): grains – 6 µm, size 4×150 mm. The eluent was CH_3CN-H_2O (90:10, v:v); the elution rate was 0.8 mL/min, 25 °C.

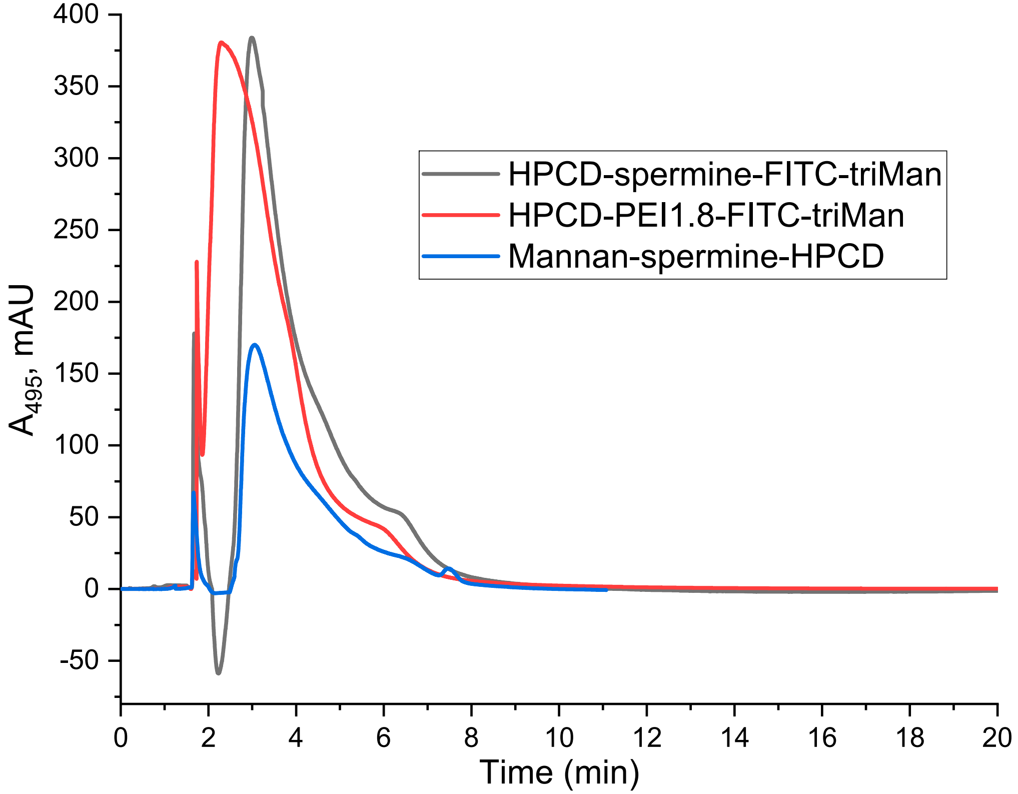

Figure S6. Dependence of the change in the intensity of the absorption band of Amide II in the FTIR spectra of ConA when binding to ligands. T = 22 °C.

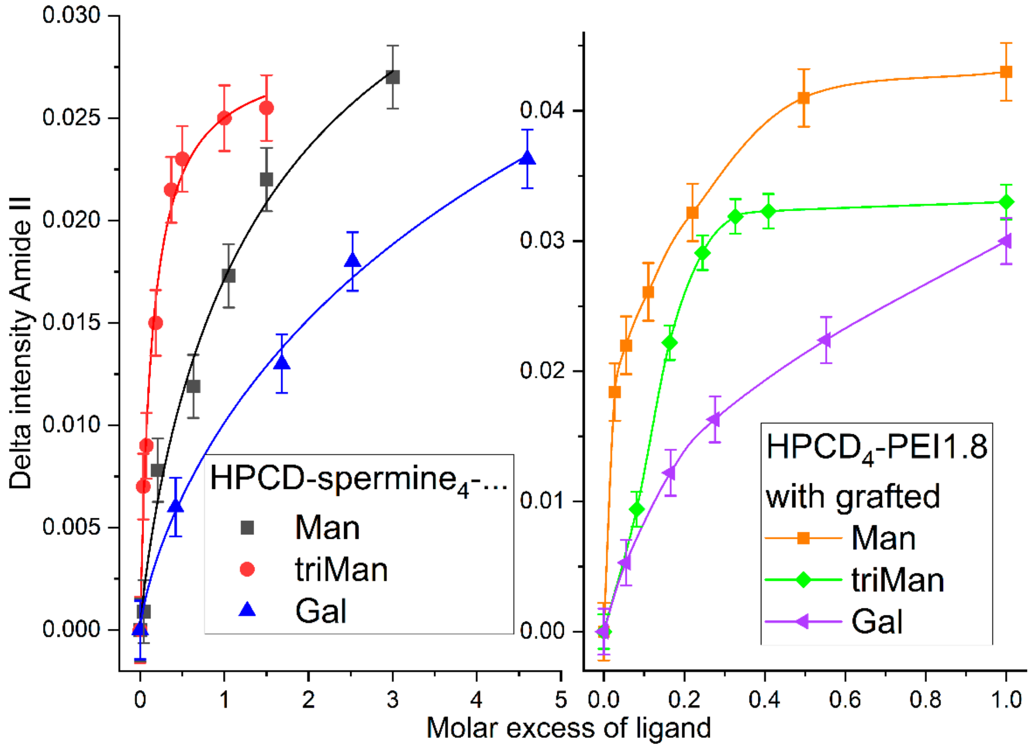

Figure S7. Amide I peak deconvolution of (a) ConA and (b) ConA complex with mannan-amCD FTIR spectra. (c) Dependences of the proportions of components in the secondary structure of ConA during ligand (mannan-amCD) binding. (d) Changes in the aggregation of ConA when binding to a ligand (mannan-amCD) relative to the native form. C_0(ConA subunit) = 0.1 mM. T = 22 °C.

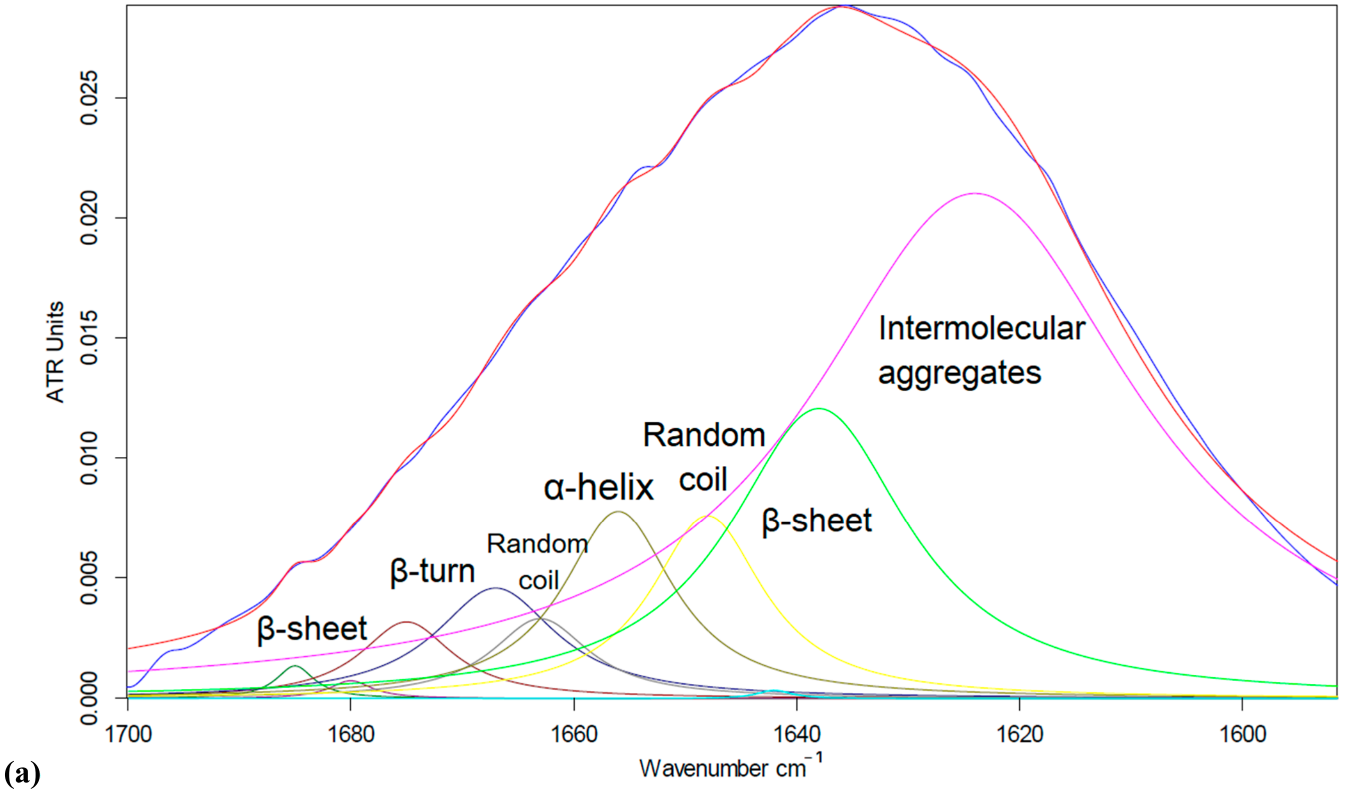

(a)

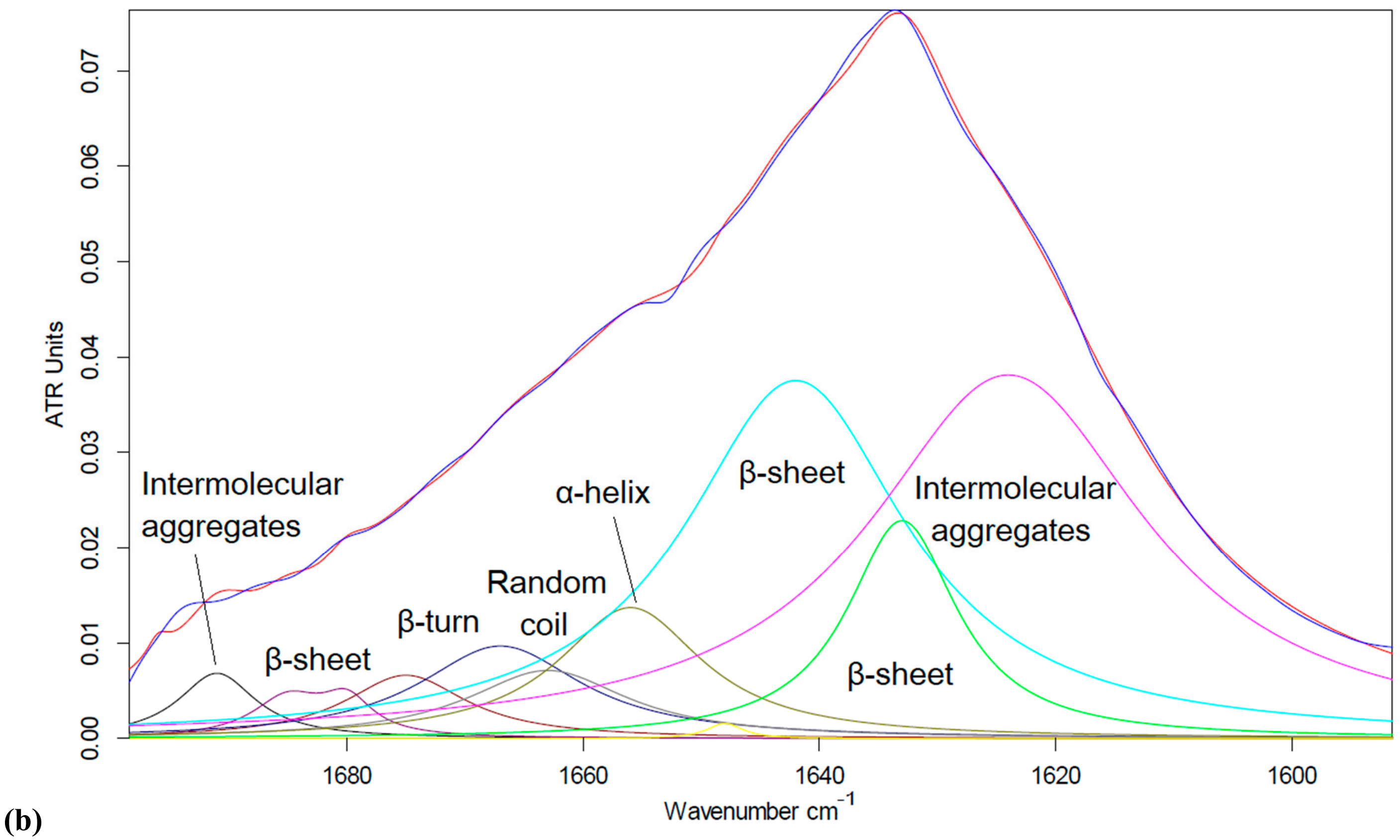

ATR Units
0.00
0.01
0.02
0.03
0.04
0.05
0.06
0.07
1680
1660
1640
1620
1600
Wavenumber cm-1
Intermolecular aggregates
β-sheet
β-turn
Random coil
α-helix
β-sheet
Intermolecular aggregates
β-sheet

(b)

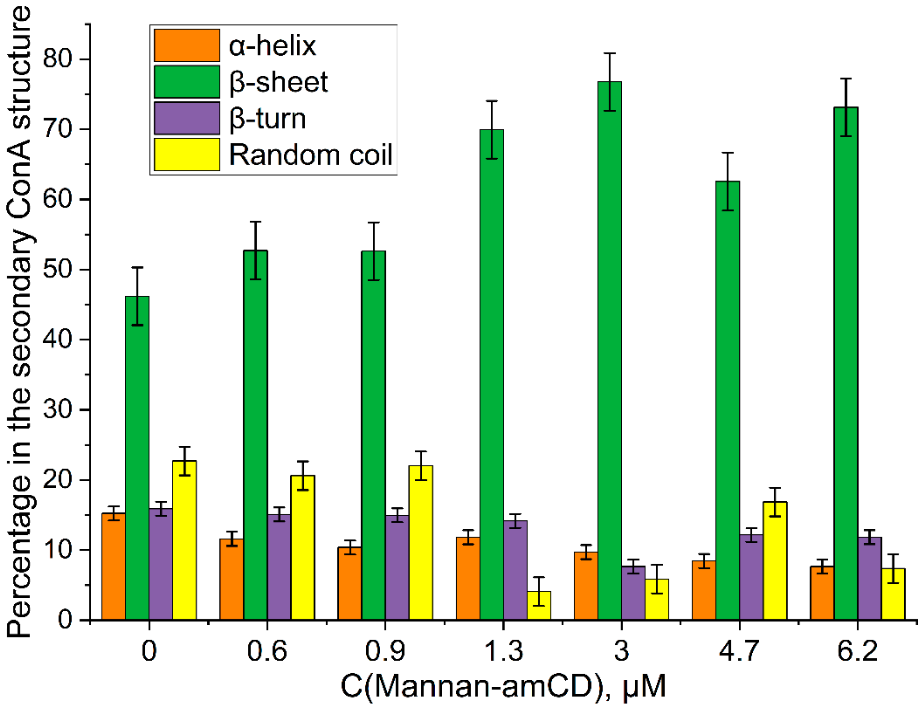

α-helix
β-sheet
β-turn
Random coil
Percentage in the secondary ConA structure
0
10
20
30
40
50
60
70
80
0
0.6
0.9
1.3
3
4.7
6.2
C(Mannan-amCD), µM

(c)

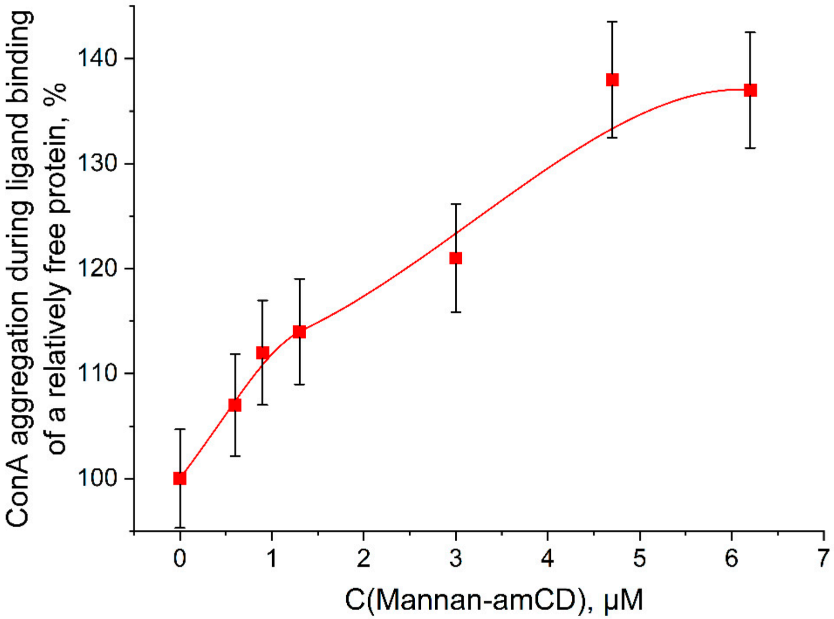

ConA aggregation during ligand binding of a relatively free protein, %
100
110
120
130
140
0
1
2
3
4
5
6
7
C(Mannan-amCD), µM

(d)

Figure S8. Fourier transform infrared spectra of **(a)** EG (eugenol) alone and its complexes with PEI1.8-triMan; **(b)** Safrole alone and its complexes with PEI1.8-triMan; **(c)** FTIR spectra – loading of four components into a molecular container at once: moxifloxacin 0.9 mM, eugenol 17 mM, safrole 12 mM, menthol 12 mM. T = 22 °C.

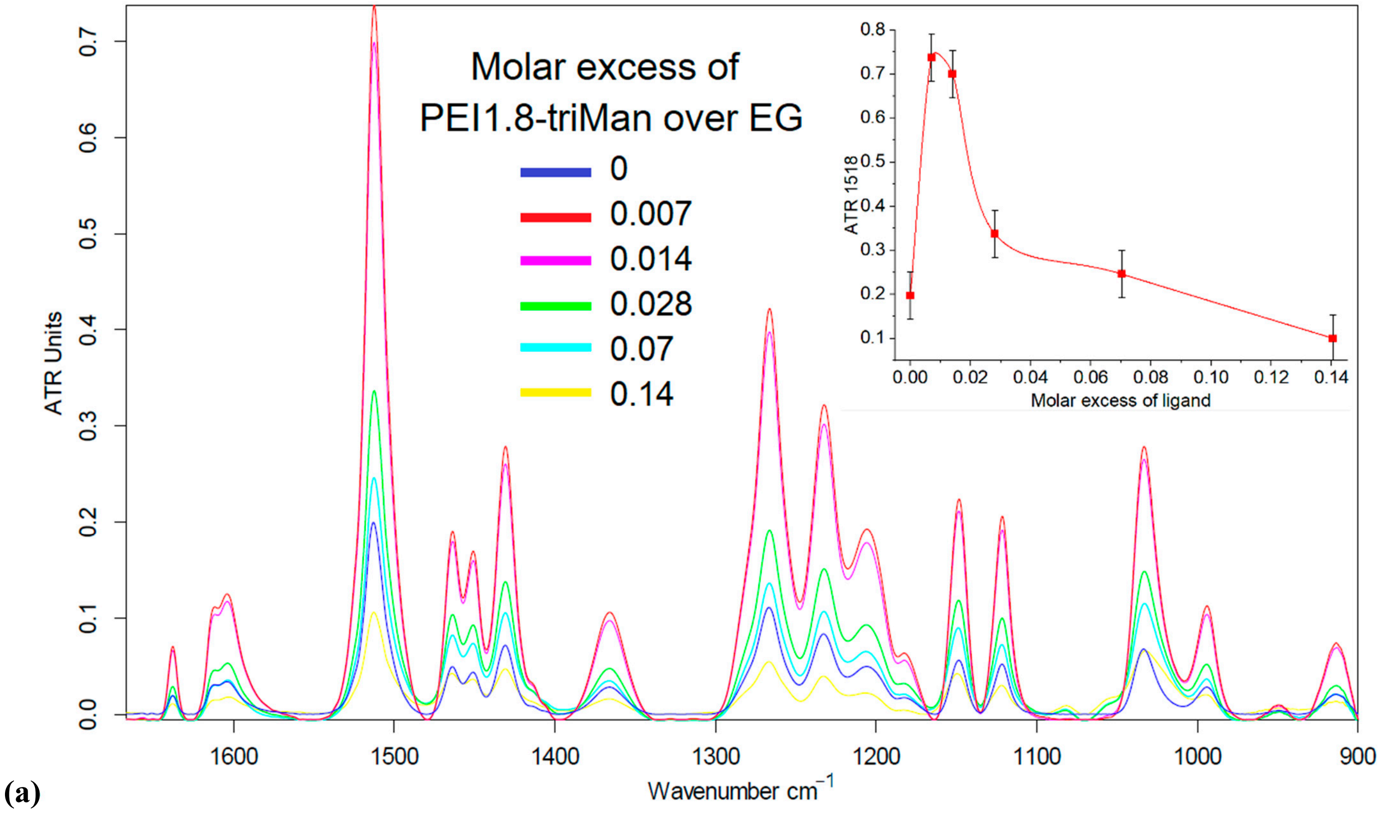

(a)

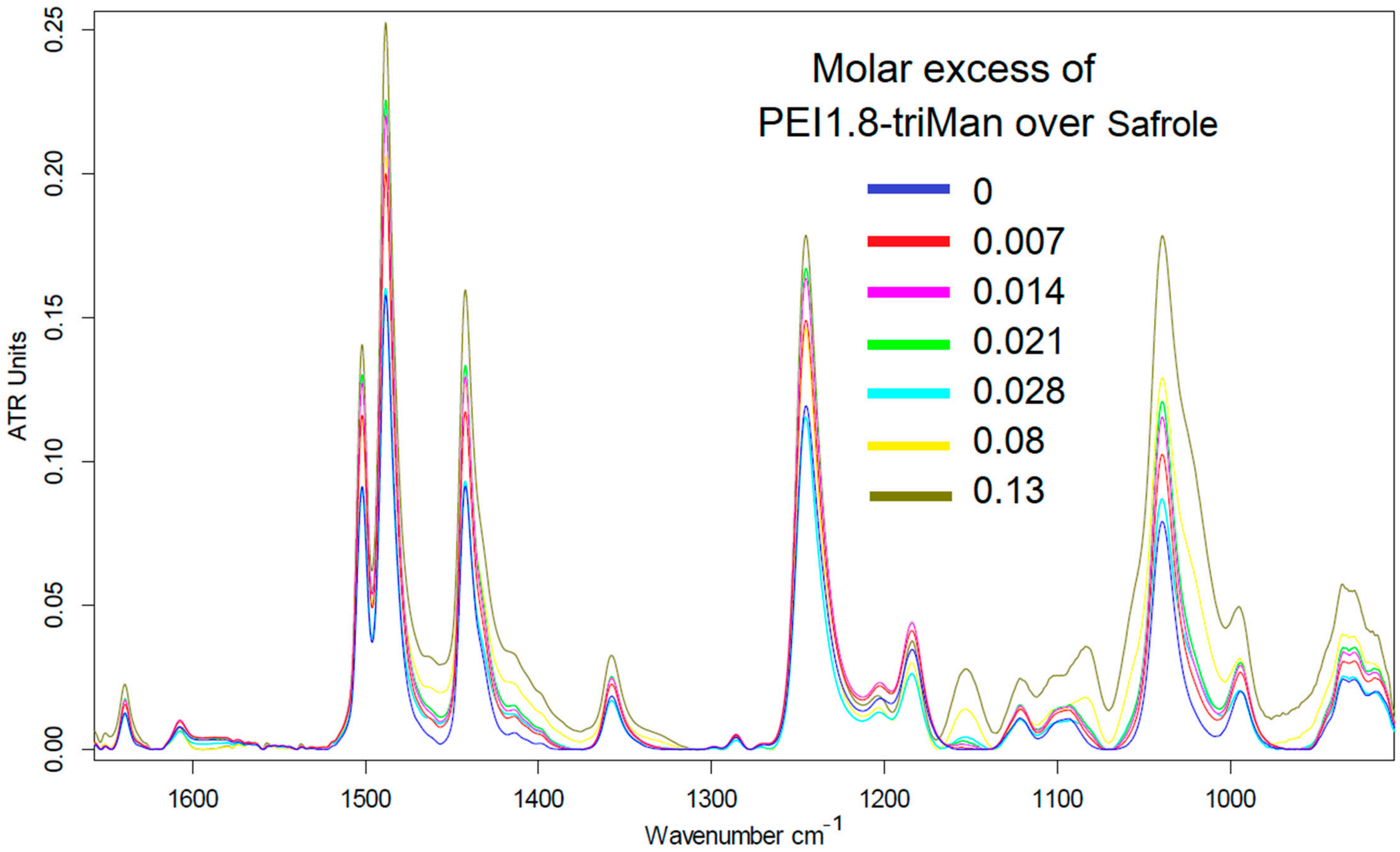
Molar excess of
PEI1.8-triMan over Safrole
0
0.007
0.014
0.021
0.028
0.08
0.13
ATR Units
0.00
0.05
0.10
0.15
0.20
0.25
1600
1500
1400
1300
1200
1100
1000
Wavenumber cm-1

(b)

(c)

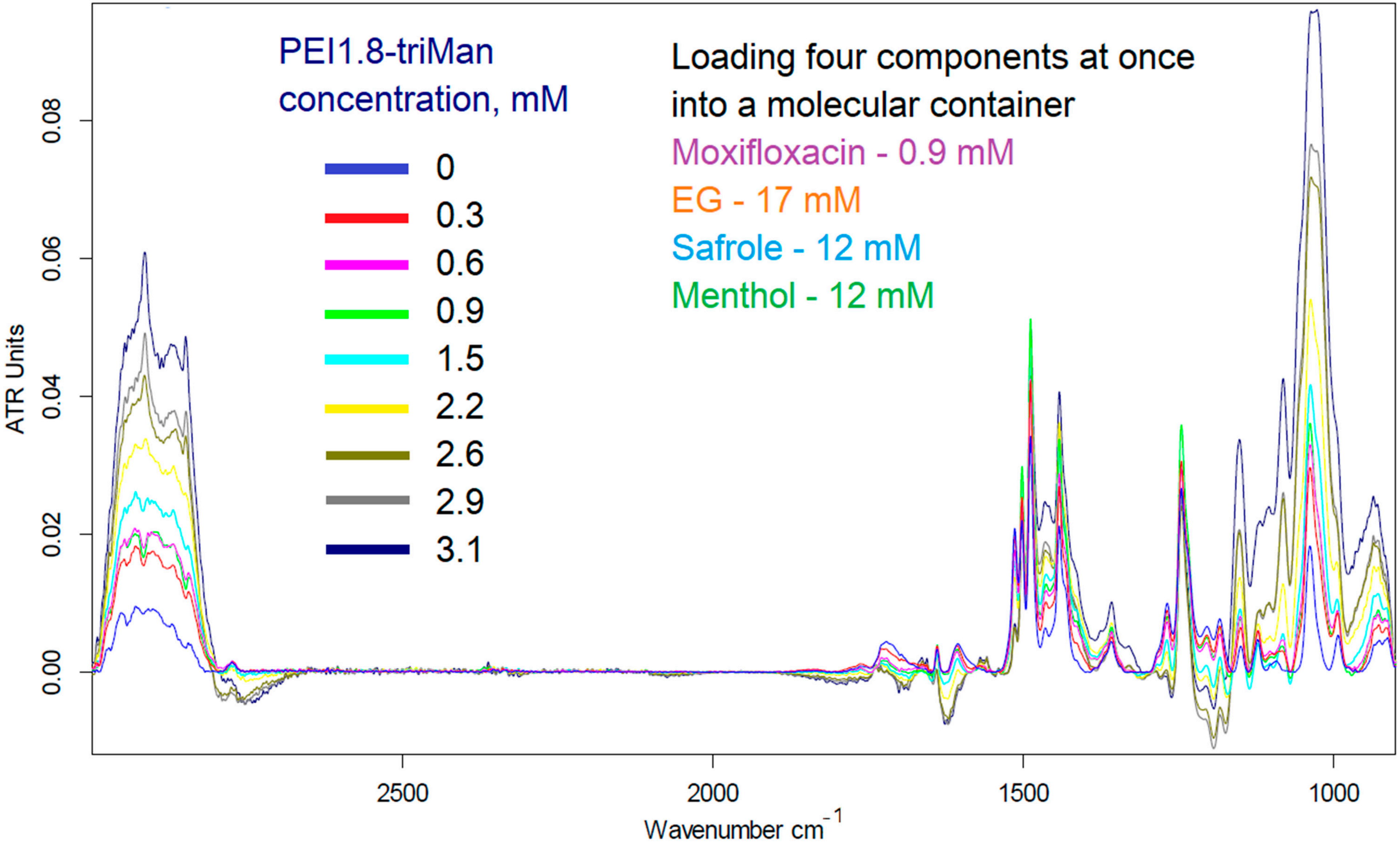
PEI1.8-triMan concentration, mM
0
0.3
0.6
0.9
1.5
2.2
2.6
2.9
3.1
Loading four components at once into a molecular container
Moxifloxacin - 0.9 mM
EG - 17 mM
Safrole - 12 mM
Menthol - 12 mM
ATR Units
0.00
0.02
0.04
0.06
0.08
2500
2000
1500
1000
Wavenumber cm-1

Figure S9. ^{1}H NMR spectra of **(a)** MF, **(b)** MF complexed with mannan-spermine-HPCD (w:w = 1:1). Proton assignment is given. D_2O, 400 MHz.

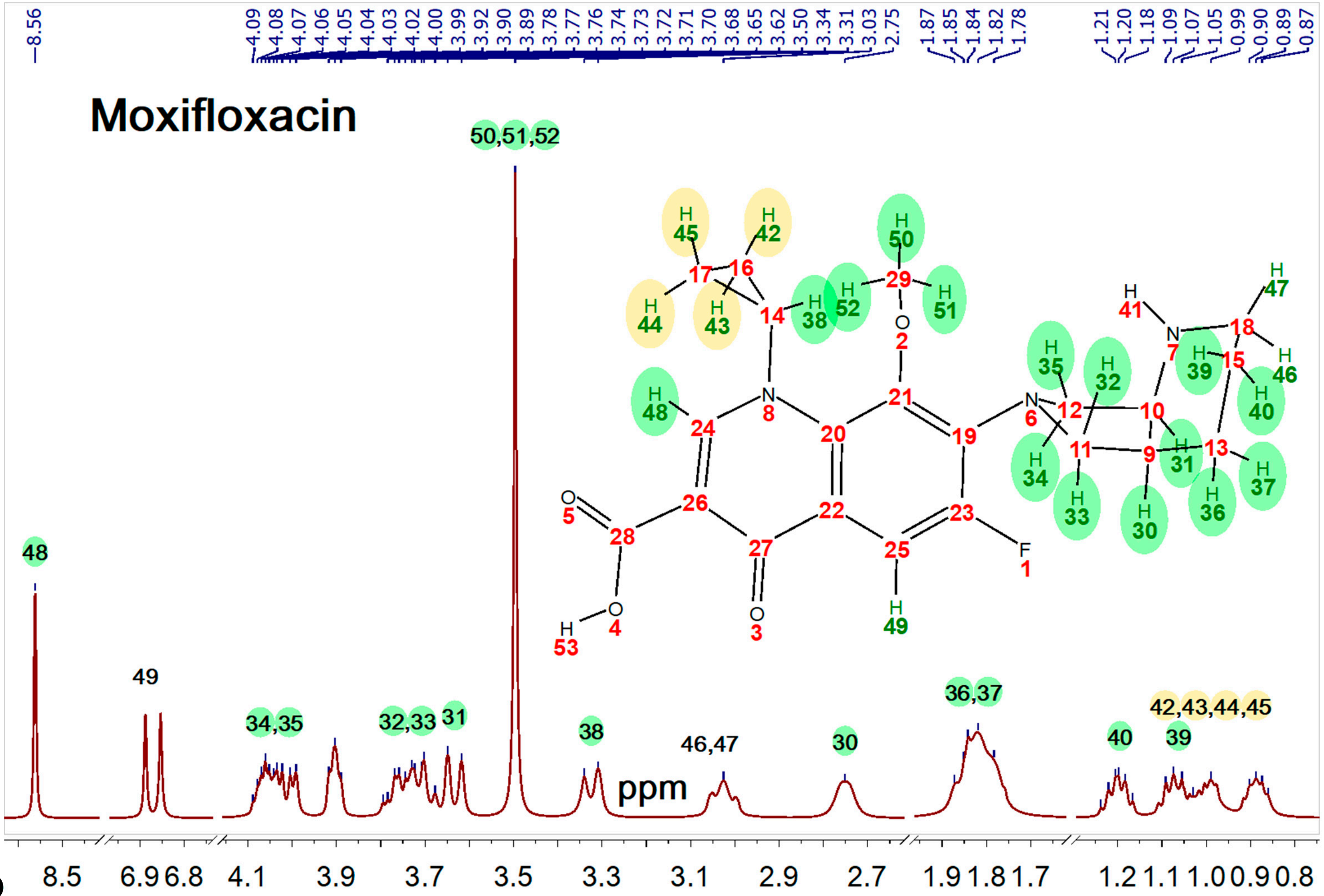

(a)

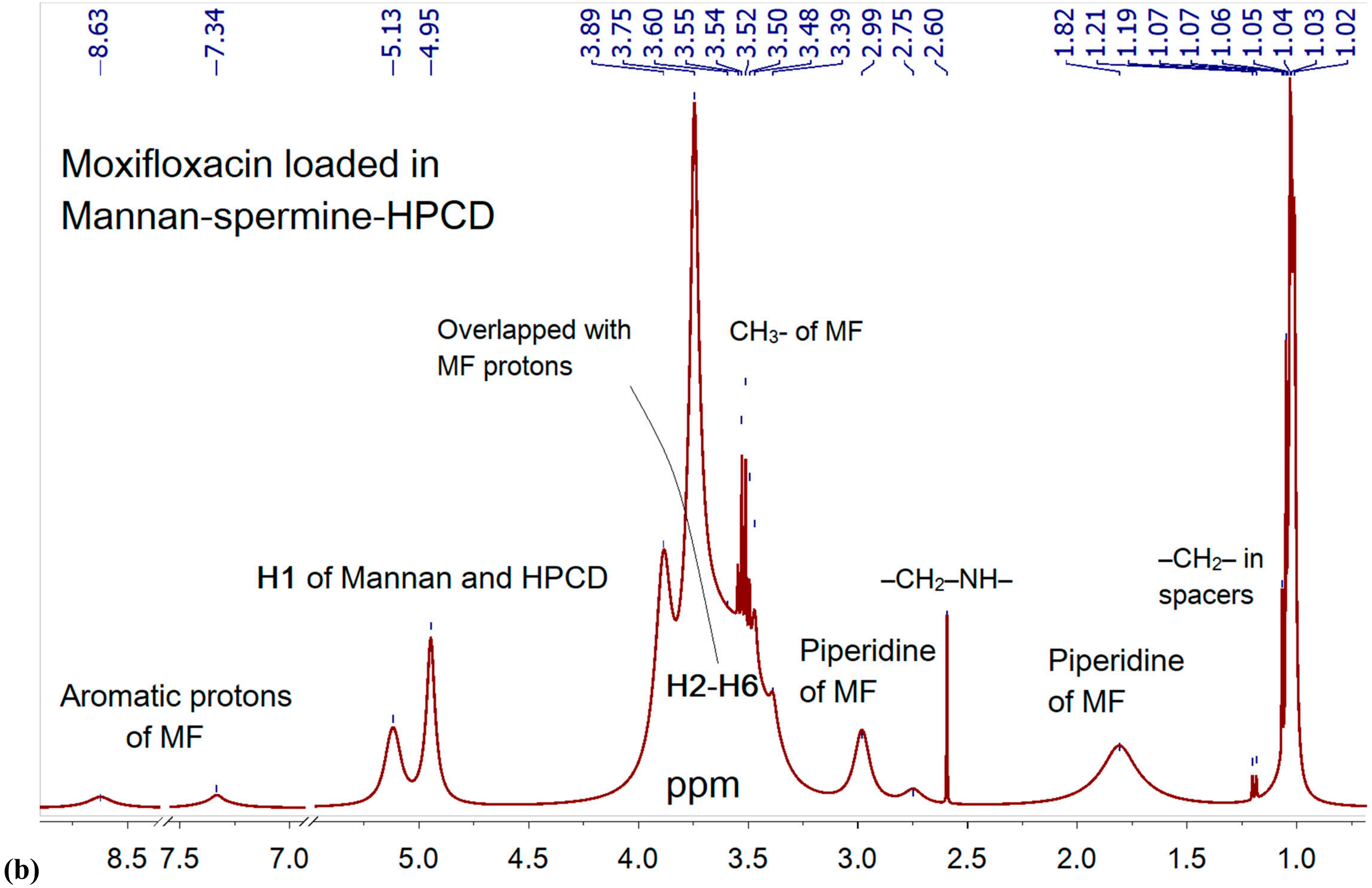

8.63
7.34
5.13
4.95
3.89
3.75
3.60
3.55
3.54
3.52
3.50
3.48
3.39
2.99
2.75
2.60
1.82
1.21
1.19
1.07
1.07
1.06
1.05
1.04
1.03
1.02
Moxifloxacin loaded in
Mannan-spermine-HPCD
Overlapped with
MF protons
CH3- of MF
H1 of Mannan and HPCD
–CH2–NH–
–CH2– in
spacers
Piperidine
of MF
Piperidine
of MF
H2-H6
Aromatic protons
of MF
ppm
8.5
7.5
7.0
5.0
4.5
4.0
3.5
3.0
2.5
2.0
1.5
1.0

(b)

Figure S10. Immunocytochemical evaluation of CD206 in THP-1 derived macrophage-like cells (red channel). Phagocytosis assay with polymers (according to table S1) at concentration №2 conjugated with a FITC fluorescent label after incubation for 40 min (green channel). Phase contrast microscopy, fluorescent microscopy, blue channel —nuclei stained with DAPI. Scale bar, 100 µm.

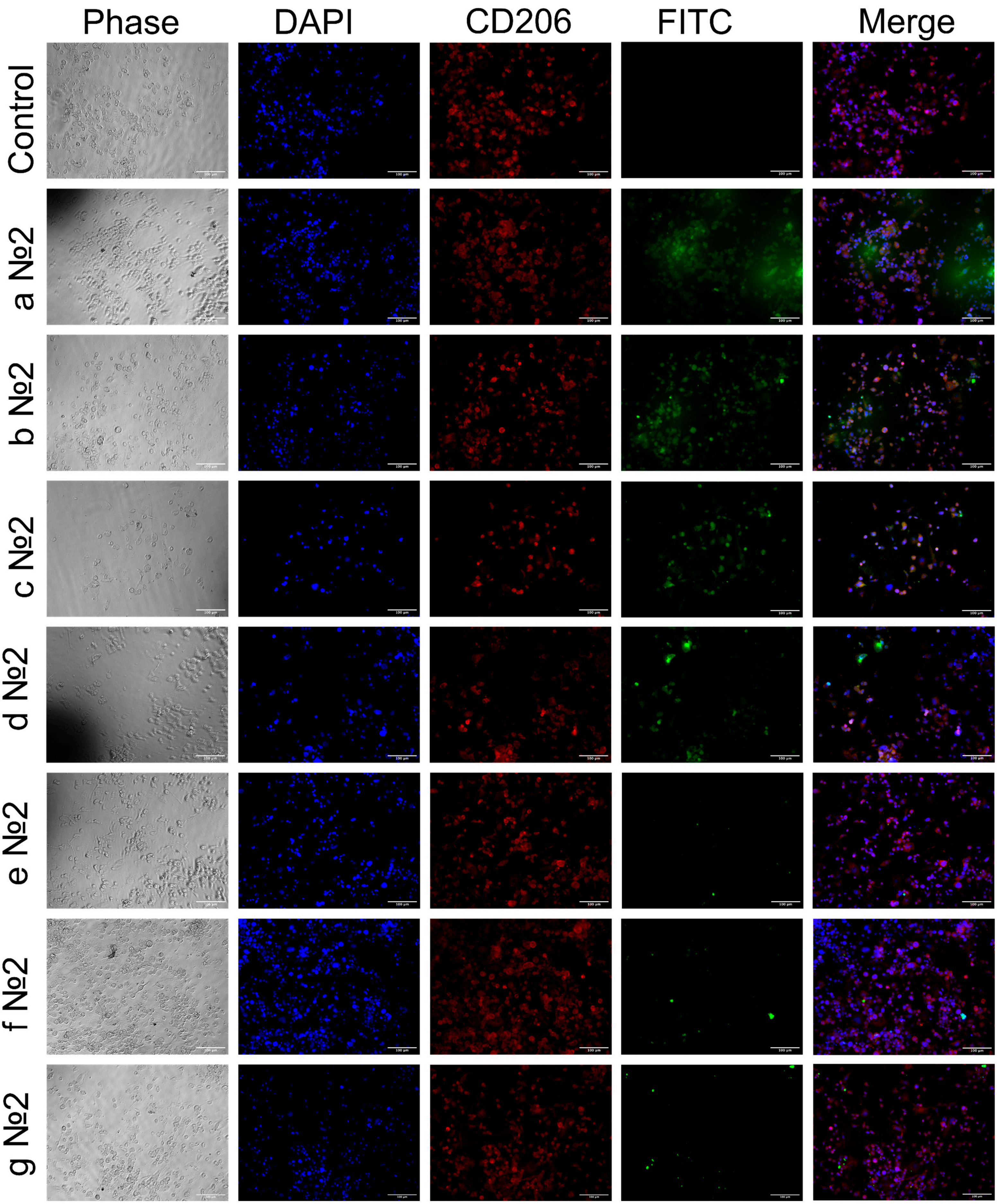

Figure S11. Flow cytometry of macrophage, gating strategy. (A) – exclusion of cellular debris based on forward and side light scattering, (B) – selection of single cells by forward scatter signal area and height, (C) – selection of single cells by side scatter signal width and height. (D,E) – overlay of a control sample (blue) and a sample after incubation with one of the polymers (red) represented by a dot plot (D) and a histogram (E). The percentage of cells in the gate is indicated under the name of the gate.

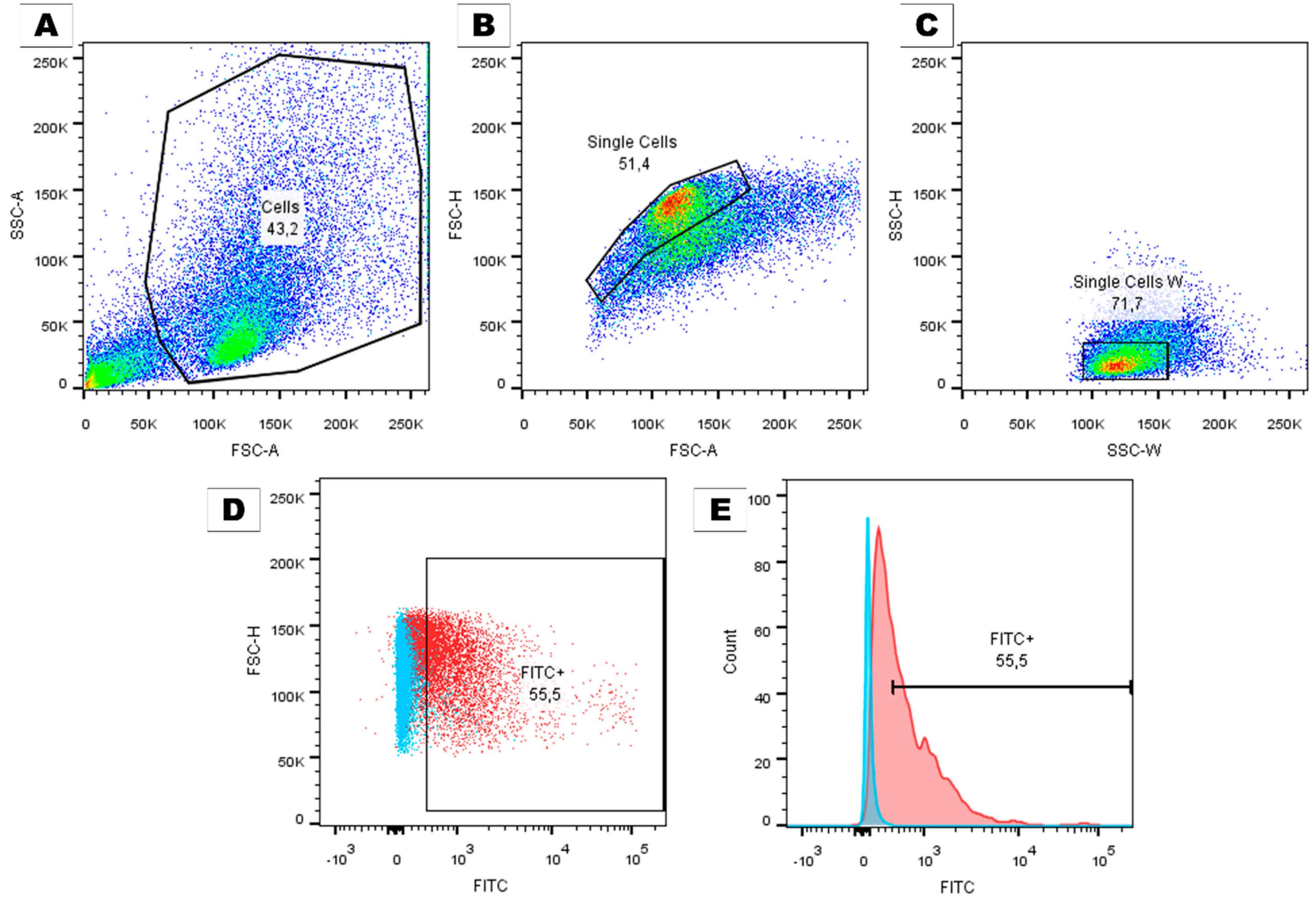

Table S1. Characteristics of polymer conjugates used for experiments with macrophages: capture efficiency, specificity – depending on the molecular architecture, ligand components and carbohydrate label.

Ligand designation	Carrier*	Label (n)**	ω(FITC), %	Final concentration for macrophages №1	Final concentration for macrophages №2
**1b**	Mannan-spermine-HPCD-FITC (1 : 15 : 10 : 2)	Mannan itself	5	0.1	0.05
**2a**	HPCD-PEI1.8-FITC* (3 : 1 : 0.5 : n)**	Man (12)	2.2	0.23	0.115
**2b**		triMan (3)			
**2c**		Gal (12)			
**3a**	HPCD-spermine-FITC (1 : 4 : 0.5 : n)**	Man (3)	8	0.065	0.0325
**3b**		triMan (1-2)			
**3c**		Gal (3)			
